# Supplementary material for: A novel histological index for evaluation of environmental enteric dysfunction identifies geographic-specific features of enteropathy among children with suboptimal growth
Source: PLoS Negl Trop Dis. 2020 Jan 13;14(1):e0007975. doi: 10.1371/journal.pntd.0007975 (PMC6980693; doi:10.1371/journal.pntd.0007975)
Supplement: S1 Checklist — (DOC) [file pntd.0007975.s001.doc]

STROBE Statement—Checklist of items that should be included in reports of ***cross-sectional studies***

|  | Item No | Recommendation |
| --- | --- | --- |
| **Title and abstract** | 1 | (*a*) Indicate the study’s design with a commonly used term in the title or the abstract  Abstract, paragraph 2 |
|  |  | (*b*) Provide in the abstract an informative and balanced summary of what was done and what was found  Abstract, paragraph 4 |
| Introduction | | |
| Background/rationale | 2 | Explain the scientific background and rationale for the investigation being reported  Introduction, paragraphs 2, 3 |
| Objectives | 3 | State specific objectives, including any prespecified hypotheses  Introduction, paragraph 4 |
| Methods | | |
| Study design | 4 | Present key elements of study design early in the paper  Methods, paragraphs 1-3 |
| Setting | 5 | Describe the setting, locations, and relevant dates, including periods of recruitment, exposure, follow-up, and data collection  Methods, paragraphs 1, 2 |
| Participants | 6 | (*a*) Give the eligibility criteria, and the sources and methods of selection of participants  Methods, paragraph 1, Table 1 |
| Variables | 7 | Clearly define all outcomes, exposures, predictors, potential confounders, and effect modifiers. Give diagnostic criteria, if applicable  Methods, paragraphs 3-6 |
| Data sources/ measurement | 8* | For each variable of interest, give sources of data and details of methods of assessment (measurement). Describe comparability of assessment methods if there is more than one group  Methods, paragraphs 7-11 |
| Bias | 9 | Describe any efforts to address potential sources of bias  Methods, paragraphs 7-11 |
| Study size | 10 | Explain how the study size was arrived at  Methods, paragraph 10 |
| Quantitative variables | 11 | Explain how quantitative variables were handled in the analyses. If applicable, describe which groupings were chosen and why  Methods, paragraphs 7-11 |
| Statistical methods | 12 | (*a*) Describe all statistical methods, including those used to control for confounding  Methods, paragraphs 7-11 |
|  |  | (*b*) Describe any methods used to examine subgroups and interactions  Methods, paragraphs 7-11 |
|  |  | (*c*) Explain how missing data were addressed  Methods, paragraphs 7-11 |
|  |  | (*d*) If applicable, describe analytical methods taking account of sampling strategy  Methods, paragraphs 7-11 |
|  |  | (*e*) Describe any sensitivity analyses  Methods, paragraphs 7-8 |
| Results | | |
| Participants | 13* | (a) Report numbers of individuals at each stage of study—eg numbers potentially eligible, examined for eligibility, confirmed eligible, included in the study, completing follow-up, and analysed  Table 2 |
|  |  | (b) Give reasons for non-participation at each stage  Not applicable |
|  |  | (c) Consider use of a flow diagram  Not applicable |
| Descriptive data | 14* | (a) Give characteristics of study participants (eg demographic, clinical, social) and information on exposures and potential confounders  Table 2 |
|  |  | (b) Indicate number of participants with missing data for each variable of interest  Results, paragraph 5 and Figure 1 |
| Outcome data | 15* | Report numbers of outcome events or summary measures  Results, paragraphs 5-7 |
| Main results | 16 | (*a*) Give unadjusted estimates and, if applicable, confounder-adjusted estimates and their precision (eg, 95% confidence interval). Make clear which confounders were adjusted for and why they were included  Results, paragraphs 9, 10 |
|  |  | (*b*) Report category boundaries when continuous variables were categorized  Results, paragraphs 9, 10 |
|  |  | (*c*) If relevant, consider translating estimates of relative risk into absolute risk for a meaningful time period  Not applicable |
| Other analyses | 17 | Report other analyses done—eg analyses of subgroups and interactions, and sensitivity analyses  Results, paragraphs 7-10 |
| Discussion | | |
| Key results | 18 | Summarise key results with reference to study objectives  Discussion, paragraph 1 |
| Limitations | 19 | Discuss limitations of the study, taking into account sources of potential bias or imprecision. Discuss both direction and magnitude of any potential bias  Discussion, paragraphs 2, 6 |
| Interpretation | 20 | Give a cautious overall interpretation of results considering objectives, limitations, multiplicity of analyses, results from similar studies, and other relevant evidence  Discussion, paragraphs 3, 4 |
| Generalisability | 21 | Discuss the generalisability (external validity) of the study results  Discussion, paragraph 9 |
| Other information | | |
| Funding | 22 | Give the source of funding and the role of the funders for the present study and, if applicable, for the original study on which the present article is based  Information for funding is provided through the submission website |

*Give information separately for exposed and unexposed groups.

**Note:** An Explanation and Elaboration article discusses each checklist item and gives methodological background and published examples of transparent reporting. The STROBE checklist is best used in conjunction with this article (freely available on the Web sites of PLoS Medicine at http://www.plosmedicine.org/, Annals of Internal Medicine at http://www.annals.org/, and Epidemiology at http://www.epidem.com/). Information on the STROBE Initiative is available at www.strobe-statement.org.
