# Supplementary material for: A novel histological index for evaluation of environmental enteric dysfunction identifies geographic-specific features of enteropathy among children with suboptimal growth
Source: PLoS Negl Trop Dis. 2020 Jan 13;14(1):e0007975. doi: 10.1371/journal.pntd.0007975 (PMC6980693; doi:10.1371/journal.pntd.0007975)
Supplement: S1 Table — (DOCX) [file pntd.0007975.s002.docx]

**Table S1.** Spearman correlation of total histologic score percent by pathologist pair.

|  | Pathologist 1 | Pathologist 2 | Pathologist 3 |
| --- | --- | --- | --- |
| Pathologist 1 | 1.00 |  |  |
| Pathologist 2 | 0.58 | 1.00 |  |
| Pathologist 3 | 0.69 | 0.74 | 1.00 |
