# Supplementary material for: A novel histological index for evaluation of environmental enteric dysfunction identifies geographic-specific features of enteropathy among children with suboptimal growth
Source: PLoS Negl Trop Dis. 2020 Jan 13;14(1):e0007975. doi: 10.1371/journal.pntd.0007975 (PMC6980693; doi:10.1371/journal.pntd.0007975)
Supplement: S2 Table — (DOCX) [file pntd.0007975.s003.docx]

**Table S2.** Median, IQR and range of histology scores, by cohort.

|  | **Histology Scores: Median (IQR)**  **Range** | | | |
| --- | --- | --- | --- | --- |
| **Parameter**  (range of possible scores) | **Pakistan** | **Zambia** | **St. Louis GSE** | **St. Louis Controls** |
| Acute (neutrophilic) inflammation (0-3) | 0 (0-0)  0-0.1 | 0 (0-0)  0-0 | 0 (0-0)  0-0 | 0 (0-0)  0-0 |
| Eosinophilic infiltration  (0-3) | 0 (0-0.1)  0-0.3 | 0 (0-0)  0-0.3 | 0 (0-0)  0-1.0 | 0 (0-0)  0-0 |
| Chronic inflammation  lamina propria ^1, 2,4^  (0-3) | 1.3 (1.2-1.9)  1.0-2.1 | 1.0 (1.3-2.0)  0.5-2.5 | 2.3 (2.0-2.3)  1.3-2.5 | 0.6 (0.3-1.3)  0.3-1.5 |
| Intraepithelial lymphocytes  (0-4) ^2,3,4,5^ | 2.6 (2.3-2.8)  1.1-3.2 | 0.7 (0.3-1.0)  0.3-1.7 | 2.7 (2.3-3.0)  2.0-3.3 | 0.3 (0.3-0.7)  0-1.0 |
| Villous architecture ^1,4^  (0-4) | 1.5 (1.1-1.7)  0.8-3.0 | 2.8 (2.0-3.7)  0-4.0 | 3.7 (3.0-4.0)  0.5-4.0 | 0.3 (0-1.5)  0-4.0 |
| Intramucosal Brunner glands ^1,2,4^  (0-3) | 0.7 (0.3-0.8)  0-2.0 | 0 (0-0.5)  0-1.7 | 2.7 (2.7-3.0)  1.7-3.0 | 0 (0-1.0)  0-1.7 |
| Foveolar cell metaplasia  (0-3) | 0 (0-0)  0-0 | 0 (0-0)  0-0.3 | 0 (0-0)  0-0.7 | 0 (0-0)  0-0 |
| Goblet cell density ^3,4,6^  (0-4) | 1.1 (0.7-1.3)  0.4-1.8 | 1.7 (1.5-2.2)  1.0-4.0 | 1.3 (1.0-1.7)  1-2.3 | 0.5 (0.3-0.7)  0-1.0 |
| Paneth cell density ^2,3,6^  (0-3) | 1.0 (0.7-1.1)  0.2-1.5 | 2.7 (2.0-3.0)  1.3-3.0 | 0.7 (0.7-1.0)  0.3-1.5 | 0 (0-0.3)  0-1.0 |
| Enterocyte injury ^3,6^  (0-3) | 0.3 (0.2-0.7)  0-0.9 | 1.0 (0.7-1.3)  0-2.0 | 0.7 (0.7-0.7)  0.3-1.0 | 0 (0-0)  0-0.7 |
| Epithelial detachment ^2,3^  (0-4) | 0.6 (0.4-0.7)  0.3-1.0 | 1.0 (0.7-1.2)  0.7-1.7 | 1.3 (0.7-1.3)  0.7-2.0 | 0.7 (0.3-1.3)  0.3-2.0 |
| Total histologic score ^1,4^  (0-37) | 9.3 (7.1-10.0)  6.1-11.7 | 11.5 (8.3-13.8)  7.3-15.3 | 15.0 (13.3-16.3)  9.7-17.3 | 2.3 (2.0-5.0)  1.7-10.3 |
| Total histologic score percent ^1,4^  (0-100) | 25.2 (19.2-27.5)  16.5-31.8 | 31.5 (23.0-37.9)  21.1-43.8 | 40.5 (36.0-45.0)  29.1-48.0 | 6.6 (5.4-14.1)  4.7-29.7 |

Abbreviations: GSE, gluten sensitive enteropathy; IQR, interquartile range

^1^ Significant differences between Pakistani and St. Louis GSE cohorts

^2^ Significant differences between Zambian and St Louis GSE cohorts

^3^ Significant differences between Pakistani and Zambian cohorts

^4^ Significant differences between St. Louis GSE & control cohorts

^5^ Significant differences between Pakistani and St. Louis control cohorts

^6^ Significant differences between Zambian and St. Louis control cohorts
