# Supplementary material for: A novel histological index for evaluation of environmental enteric dysfunction identifies geographic-specific features of enteropathy among children with suboptimal growth
Source: PLoS Negl Trop Dis. 2020 Jan 13;14(1):e0007975. doi: 10.1371/journal.pntd.0007975 (PMC6980693; doi:10.1371/journal.pntd.0007975)
Supplement: S3 Table — (DOCX) [file pntd.0007975.s004.docx]

**Table S3.** Marginal associations with minimal effect sizes for regression analyses of histology score parameters with patient characteristics.

| **Biomarker** | **Histologic score**  **parameter** | **Effect size**  **(coefficient**  ß**)** | **95% confidence**  **interval** | **P-value** |
| --- | --- | --- | --- | --- |
| History of diarrhea | Intramucosal Brunner glands | 0.2 | -0.02, 0.3 | 0.09 |
|  | Epithelial detachment | 0.2 | -0.03, 0.4 | 0.08 |
|  | Total histologic score percent | 0.01 | -0.002, 0.02 | 0.09 |
| Diarrhea duration | Epithelial detachment | 0.8 | -0.04, 1.6 | 0.06 |
| Hemoglobin | Intramucosal Brunner glands | -0.7 | -1.6, 0.1 | 0.09 |
| CRP | Total histologic score percent | -0.07 | -0.1, 0.003 | 0.06 |
| Lactulose:rhamnose  ratio | Enterocyte injury | 0.4 | -0.02, 0.8 | 0.06 |

Abbreviations: CRP, C-reactive protein
