# Supplementary material for: A novel histological index for evaluation of environmental enteric dysfunction identifies geographic-specific features of enteropathy among children with suboptimal growth
Source: PLoS Negl Trop Dis. 2020 Jan 13;14(1):e0007975. doi: 10.1371/journal.pntd.0007975 (PMC6980693; doi:10.1371/journal.pntd.0007975)
Supplement: S4 Table — (DOCX) [file pntd.0007975.s005.docx]

**Table S4.** Histology scores of Pakistan celiac case compared to Pakistan & St. Louis GSE cohorts histology score medians

| **Parameter**  (range of possible scores) | **Pakistan celiac case** | **Pakistan cohort**  **median (IQR)**  **range** | **St. Louis Celiac cohort**  **median (IQR)**  **range** |
| --- | --- | --- | --- |
| Acute (neutrophilic) inflammation (0-3) | 0 | 0 (0-0)  0-0.1 | 0 (0-0)  0-0 |
| Eosinophilic infiltration  (0-3) | 0 | 0 (0-0.1)  0-0.3 | 0 (0-0)  0-1.0 |
| Chronic inflammation lamina propria  (0-3) | 1.8 | 1.3 (1.2-1.9)  1.0-2.1 | 2.3 (2.0-2.3)  1.3-2.5 |
| Intraepithelial lymphocytes  (0-4) | 3.1 | 2.6 (2.3-2.8)  1.1-3.2 | 2.7 (2.3-3.0)  2.0-3.3 |
| Villous architecture  (0-4) | 1.6 | 1.5 (1.1-1.7)  0.8-3.0 | 3.7 (3.0-4.0)  0.5-4.0 |
| Intramucosal Brunner glands  (0-3) | 0.3 | 0.7 (0.3-0.8)  0-2.0 | 2.7 (2.7-3.0)  1.7-3.0 |
| Foveolar cell metaplasia  (0-3) | 0 | 0 (0-0)  0-0 | 0 (0-0)  0-0.7 |
| Goblet cell density  (0-4) | 1.8 | 1.1 (0.7-1.3)  0.4-1.8 | 1.3 (1.0-1.7)  1-2.3 |
| Paneth cell density  (0-3) | 1.1 | 1.0 (0.7-1.1)  0.2-1.5 | 0.7 (0.7-1.0)  0.3-1.5 |
| Enterocyte injury  (0-3) | 0.9 | 0.3 (0.2-0.7)  0-0.9 | 0.7 (0.7-0.7)  0.3-1.0 |
| Epithelial detachment  (0-4) | 0.9 | 0.6 (0.4-0.7)  0.3-1.0 | 1.3 (0.7-1.3)  0.7-2.0 |
| Total histologic score  (0-37) | 11.4 | 9.3 (7.1-10.0)  6.1-11.7 | 15.0 (13.3-16.3)  9.7-17.3 |
| Total histologic score percent  (0-100) | 30.9 | 25.2 (19.2-27.5)  16.5-31.8 | 40.5 (36.0-45.0)  29.1-48.0 |

Abbreviations: GSE, gluten sensitivity enteropathy; IQR, interquartile range

Note: Pakistan cohort and Pakistan celiac case scores are presented as an average for an individual child based on >1 slide image per child with each slide image scored independently.
