# Supplementary material for: A novel histological index for evaluation of environmental enteric dysfunction identifies geographic-specific features of enteropathy among children with suboptimal growth
Source: PLoS Negl Trop Dis. 2020 Jan 13;14(1):e0007975. doi: 10.1371/journal.pntd.0007975 (PMC6980693; doi:10.1371/journal.pntd.0007975)
Supplement: S1 Atlas — (PDF) [file pntd.0007975.s006.pdf]

DUODENAL HISTOPATHOLOGY GRADING SCHEME VERSION 5.5

Environmental Enteric Dysfunction Biopsy Initiative Consortium

Updated November 25, 2019

VILLUS ARCHITECTURE
GRADE 0

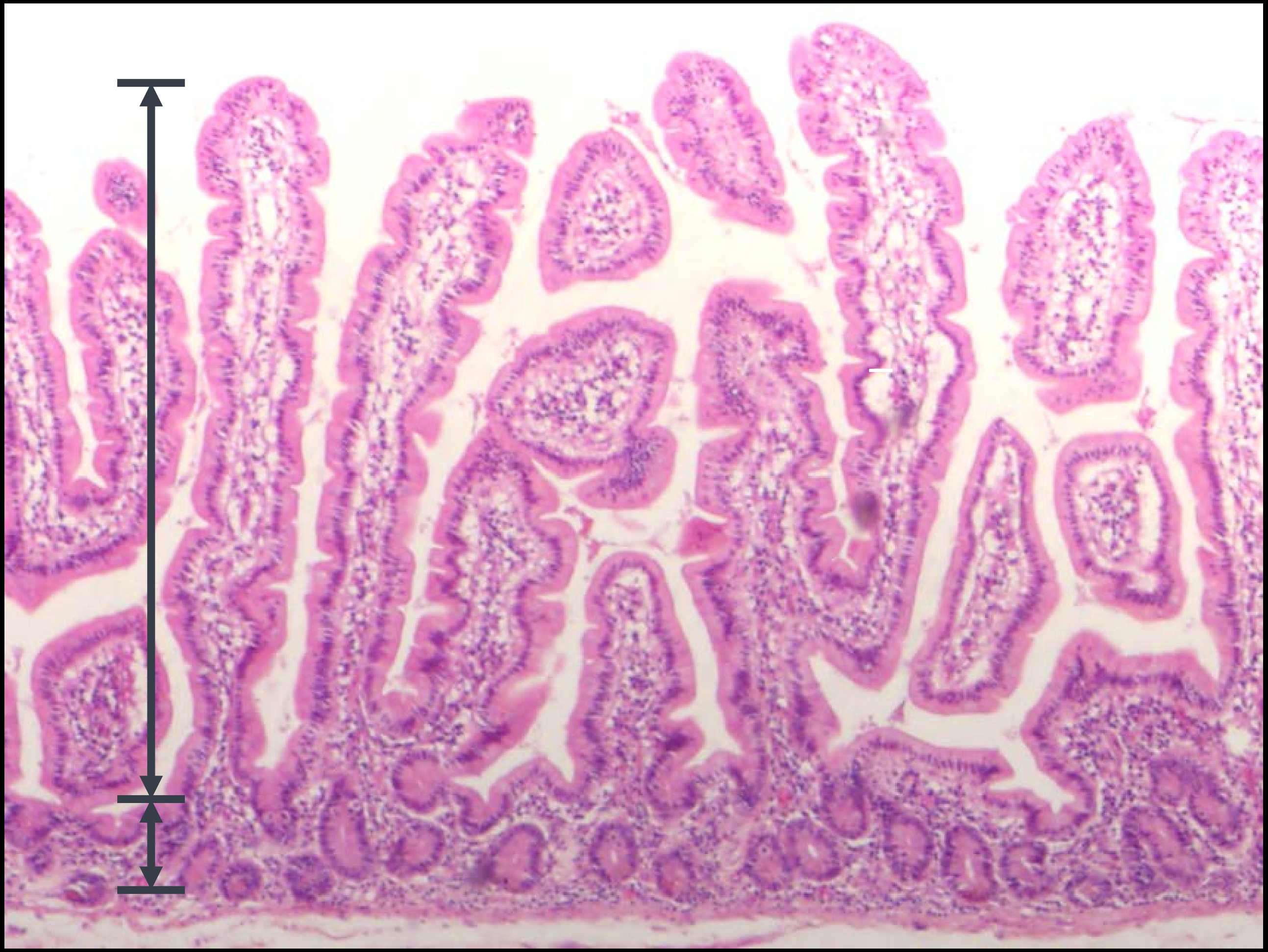

Grade 0: Majority of villi are >3 crypt lengths long

VILLUS ARCHITECTURE GRADES 1 & 2

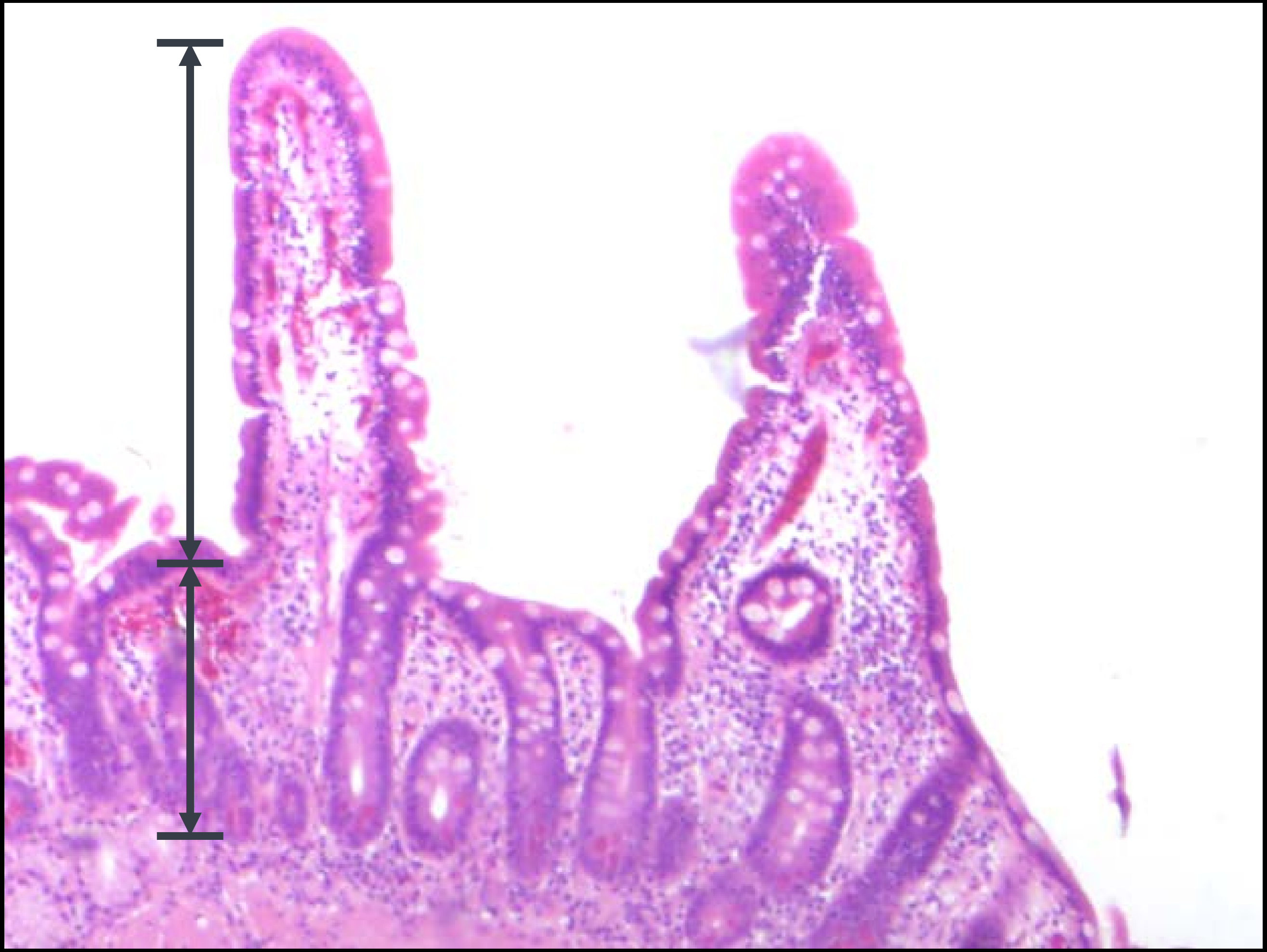

Grade 1: Villi are ≤ 3 but > 1 crypt lengths long, in ≤ 50% of mucosa

Grade 2: Villi are ≤ 3 but > 1 crypt lengths long, in > 50% of mucosa

VILLUS ARCHITECTURE GRADES 3 & 4

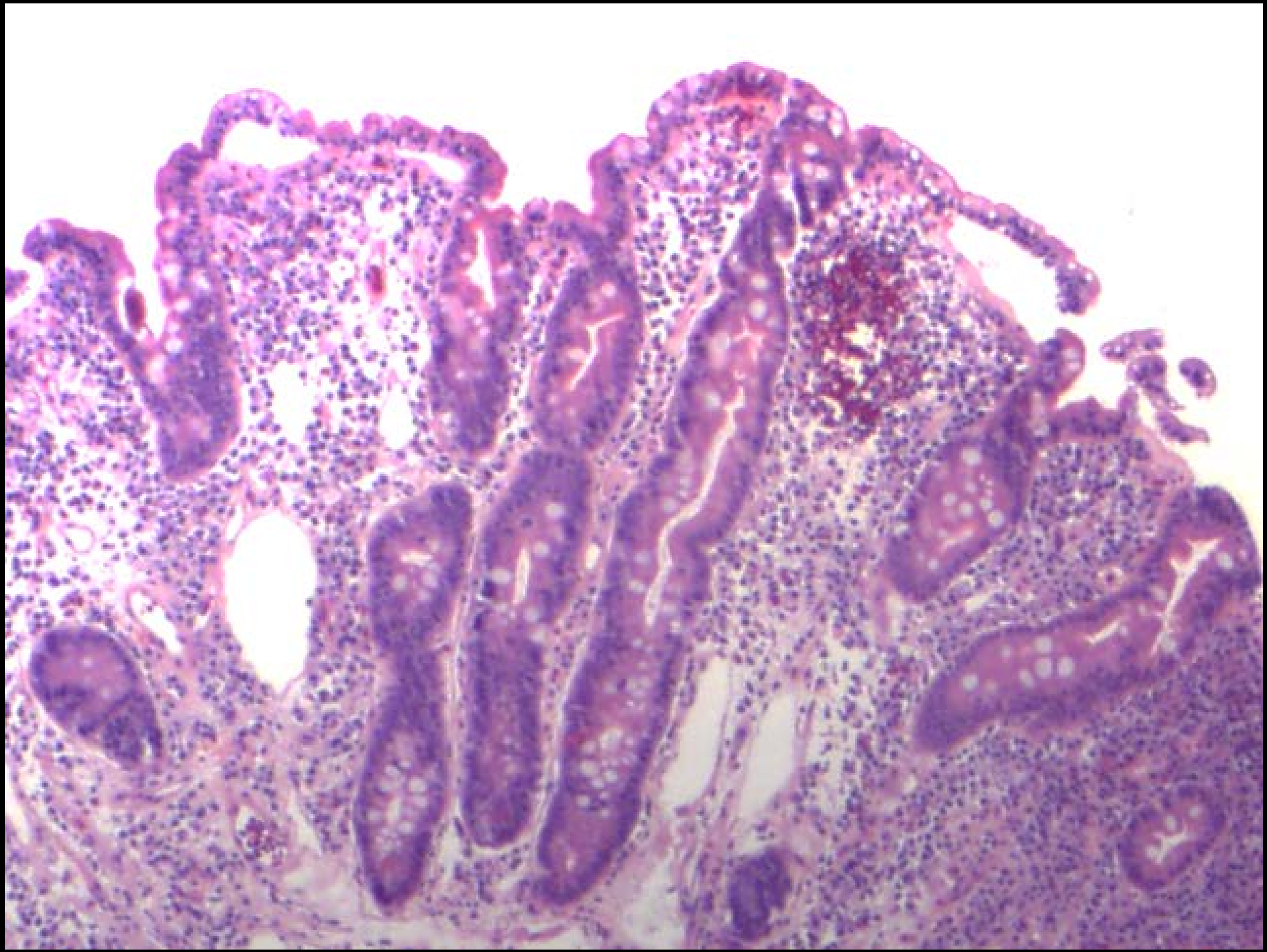

Grade 3: Villi absent, or < 1 crypt length long, in ≤ 50% of mucosa

Grade 4: Villi absent, or < 1 crypt length long, in > 50% of mucosa

GESTALT ARCHITECTURE GRADE 0

Grade 0: Majority of villi appear normal (non-blunted)

GESTALT ARCHITECTURE GRADES 1&2

Grade 1: Majority of villi show mild to moderate blunting
Grade 2: Majority of villi show moderate to severe blunting

GESTALT ARCHITECTURE
GRADE 3

Grade 3: Villus architecture is effaced (no villi observed)

EPITHELIAL DETACHMENT GRADE 0

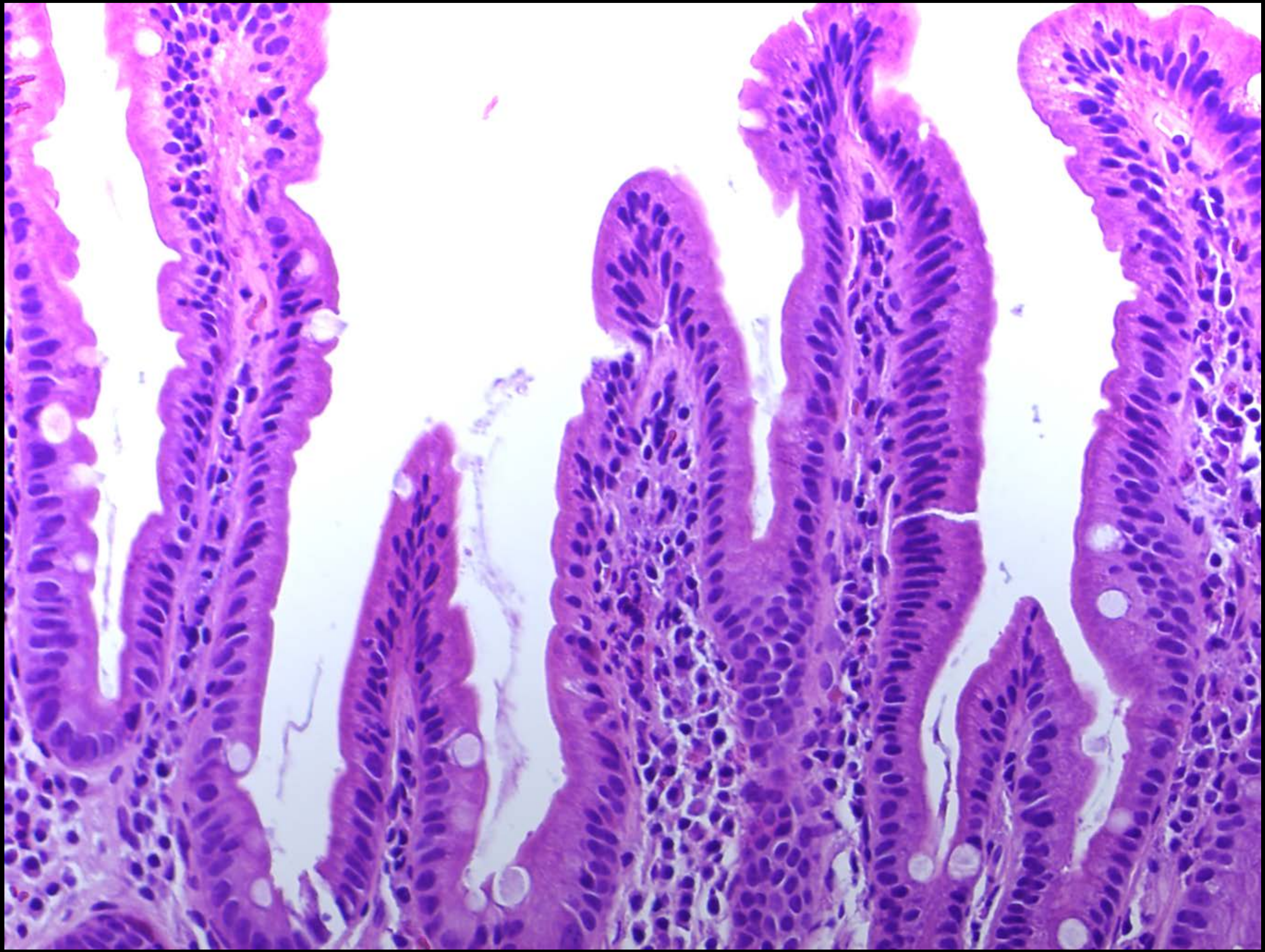

Complete coverage of mucosa by epithelial cells.

EPITHELIAL DETACHMENT GRADES 1-4

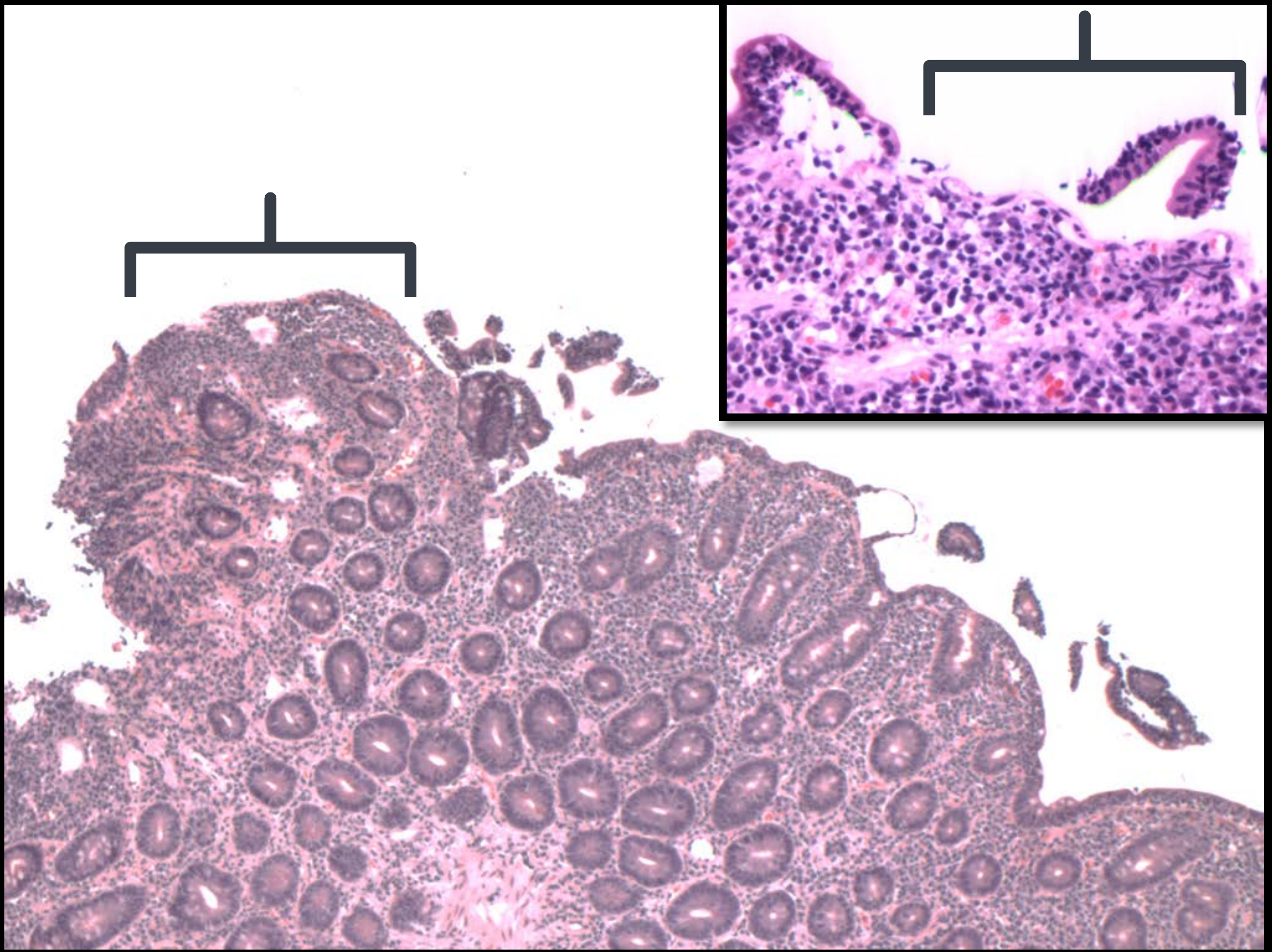

Surface epithelium missing or detached.

Grade 1: <25% of mucosa involved

Grade 2: 25-50% of mucosa involved

Grade 3: 51-75% of mucosa involved

Grade 4: >75% of mucosa involved

INTRAMUCOSAL BRUNNER GLANDS GRADE 0

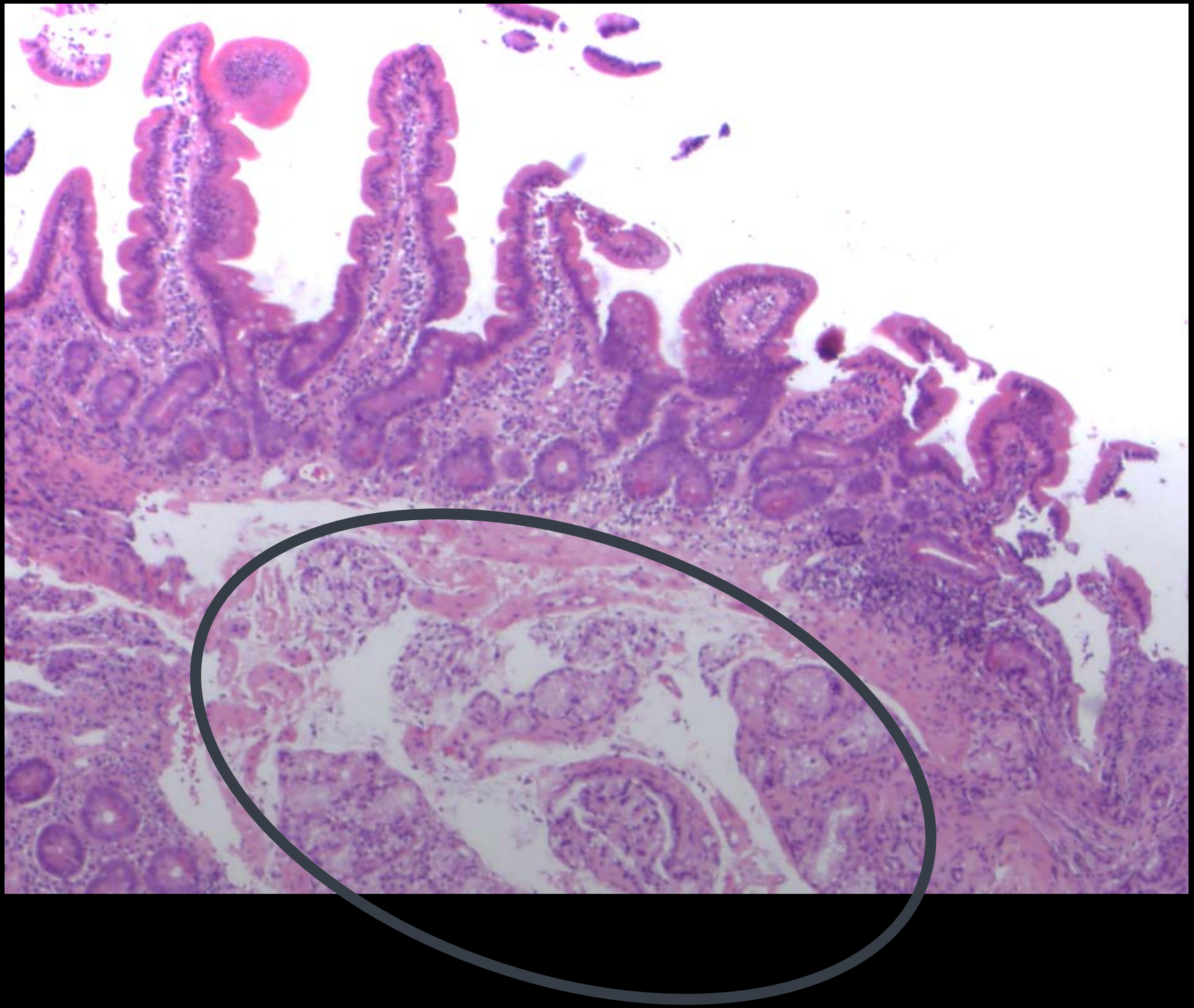

Brunner glands are in submucosa, but are not observed above the muscularis mucosae

INTRAMUCOSAL BRUNNER GLANDS GRADES 1 & 2

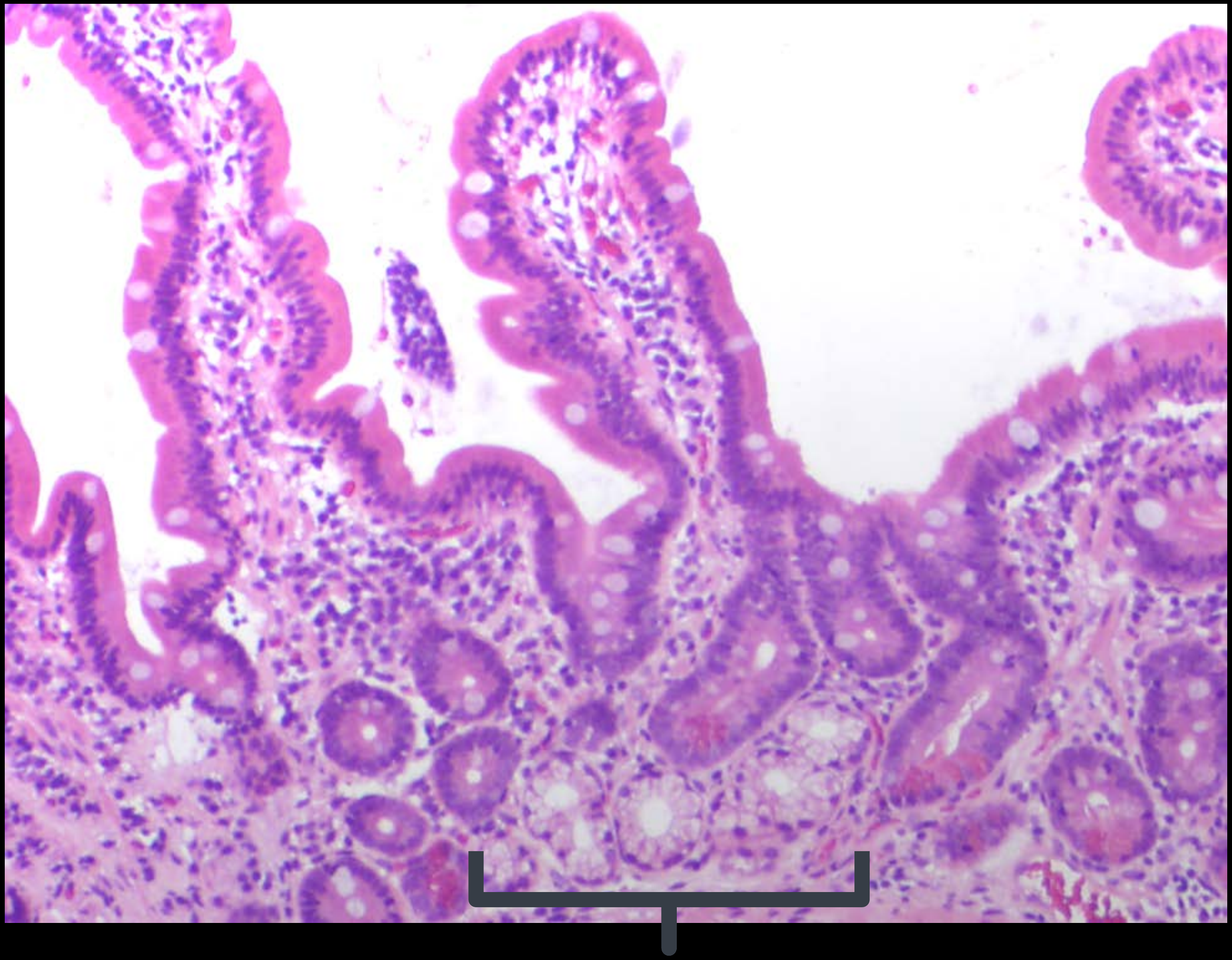

Grade 1: 1 or 2 foci of intramucosal Brunner glands, none involving more than 5 crypts

Grade 2: 3 -5 foci of intramucosal Brunner glands, none involving more than 5 crypts

INTRAMUCOSAL BRUNNER GLANDS GRADE 3

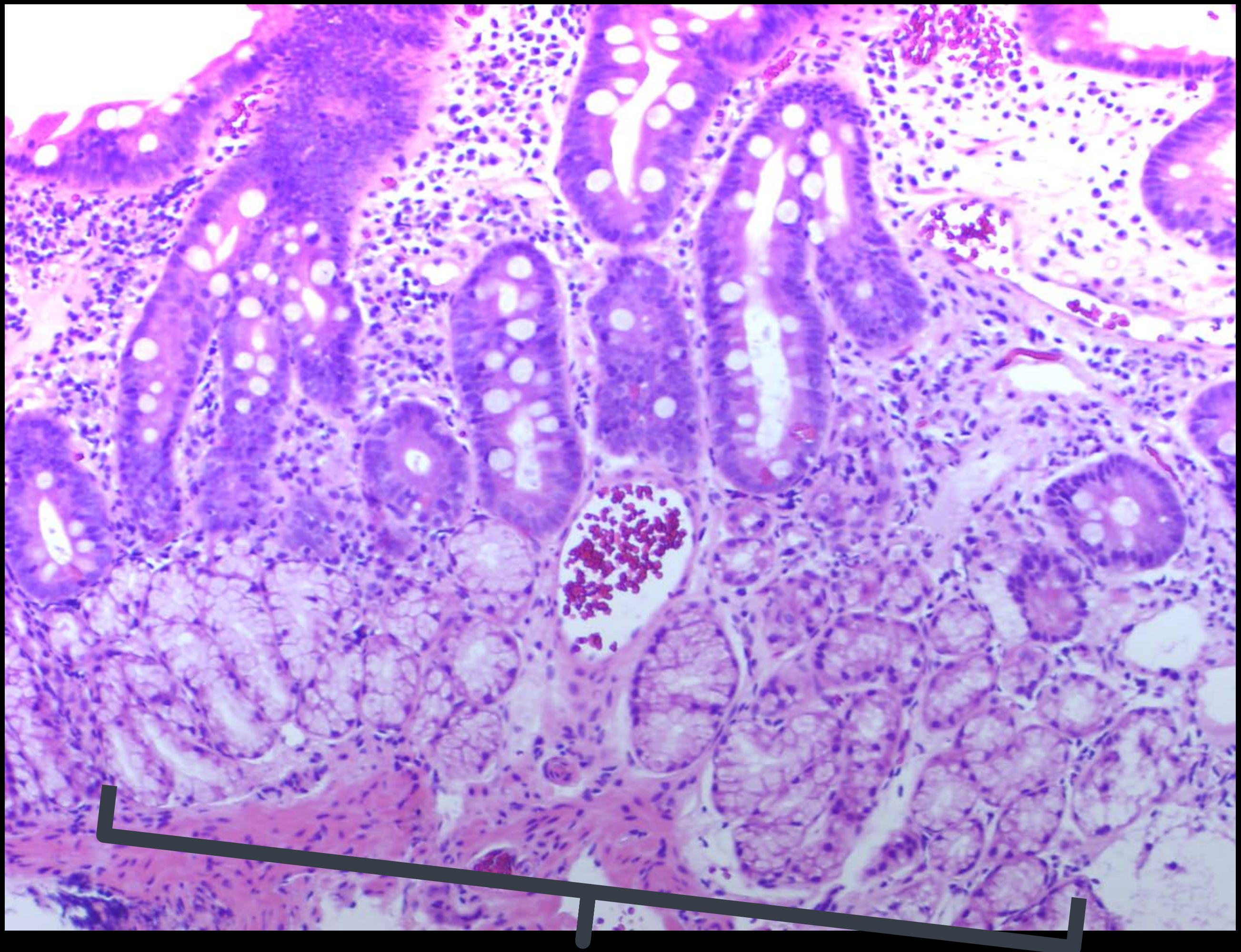

More than 5 foci of intramucosal Brunner glands, or any area involving more than 5 crypts

GOBLET CELL DENSITY GRADE 0

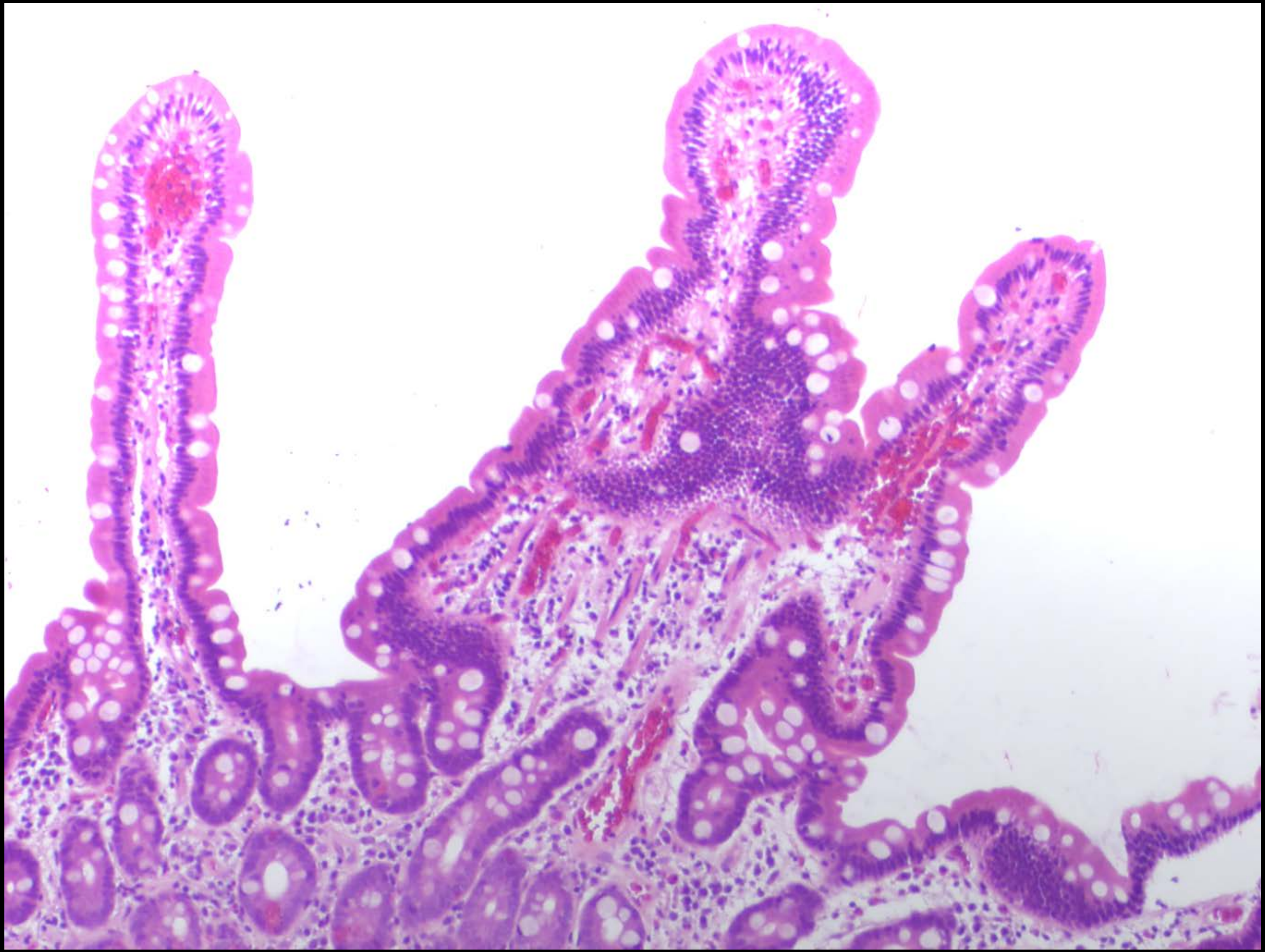

Grade 0: Normal goblet cell density (at least 1 goblet cell per 20 enterocytes) in all evaluable mucosal epithelial layer

GOBLET CELL DENSITY GRADES 1 - 4

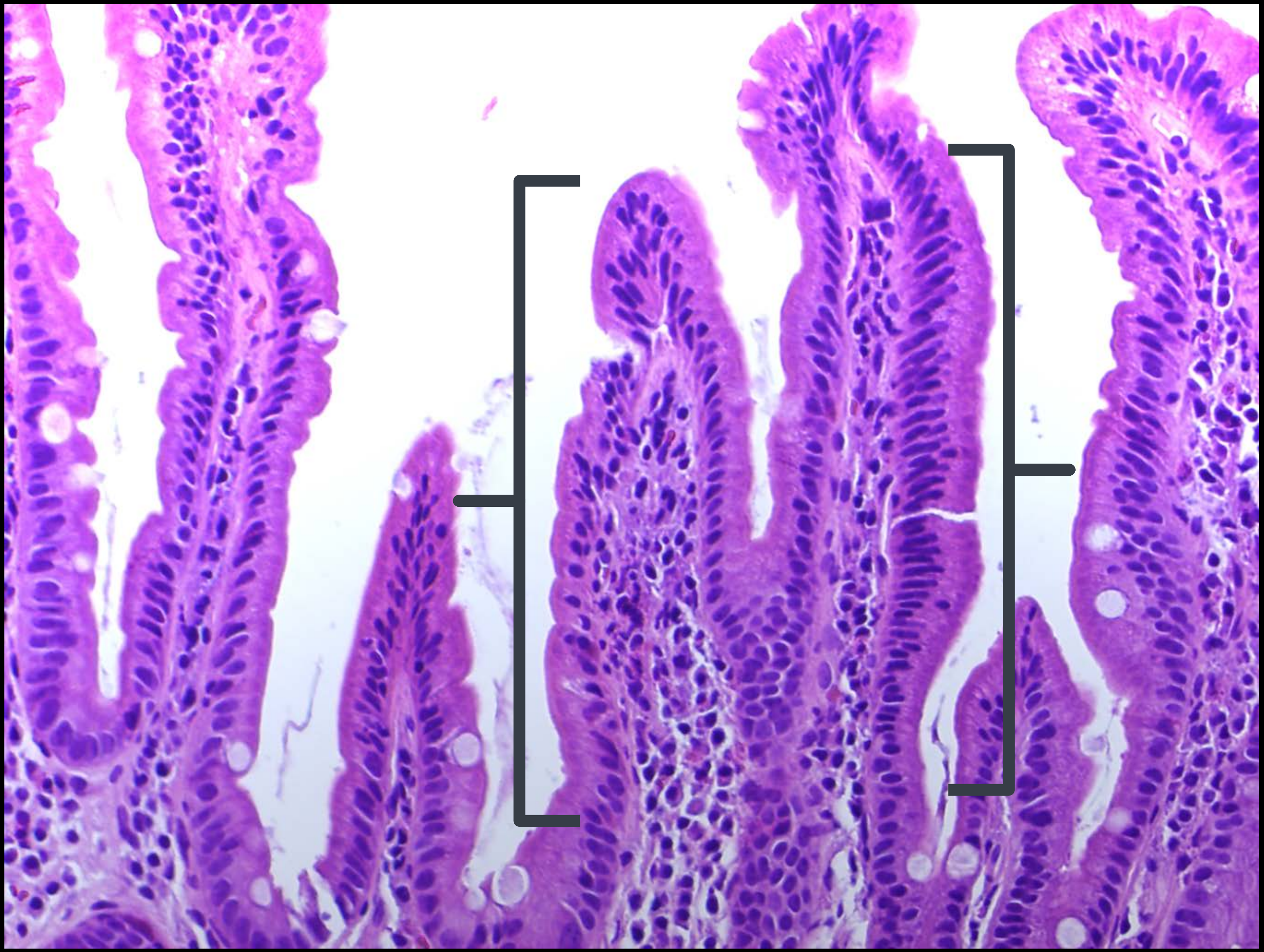

Grade 1: Decreased goblet cells (< 1/20 enterocytes) in 1-25% of evaluable mucosal epithelium
Grade 2: Decreased goblet cells (< 1/20 enterocytes) in 26-50% of evaluable mucosal epithelium
Grade3: Decreased goblet cells (< 1/20 enterocytes) in 51-75% of evaluable mucosal epithelium
Grade 4: Decreased goblet cells (< 1/20 enterocytes) in 76-100% of evaluable mucosal epithelium

FOVEOLAR CELL METAPLASIA GRADE 0

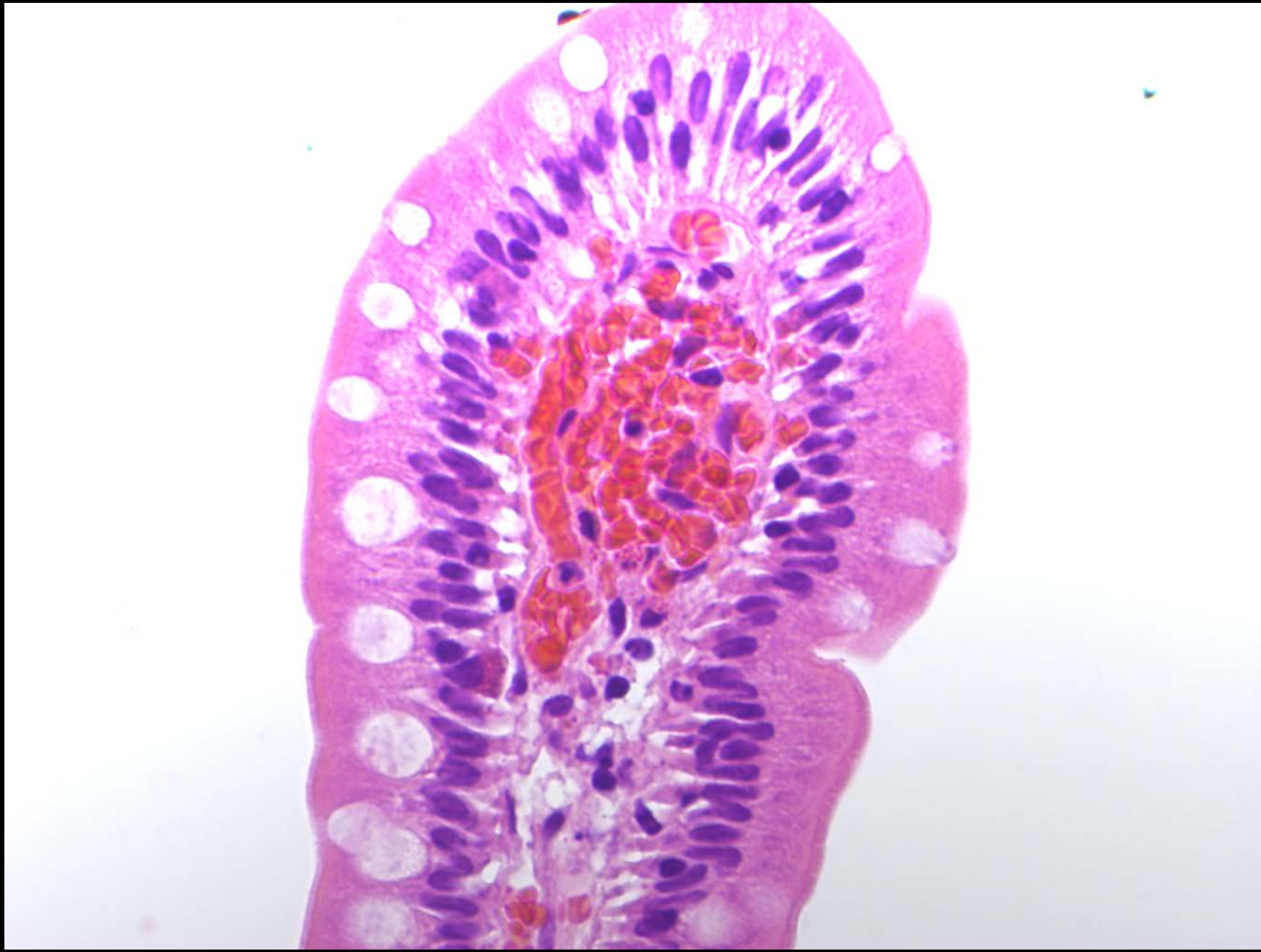

Only absorptive enterocytes and goblet cells observed on villi, no evidence of foveolar cells

FOVEOLAR CELL METAPLASIA GRADES 1-3

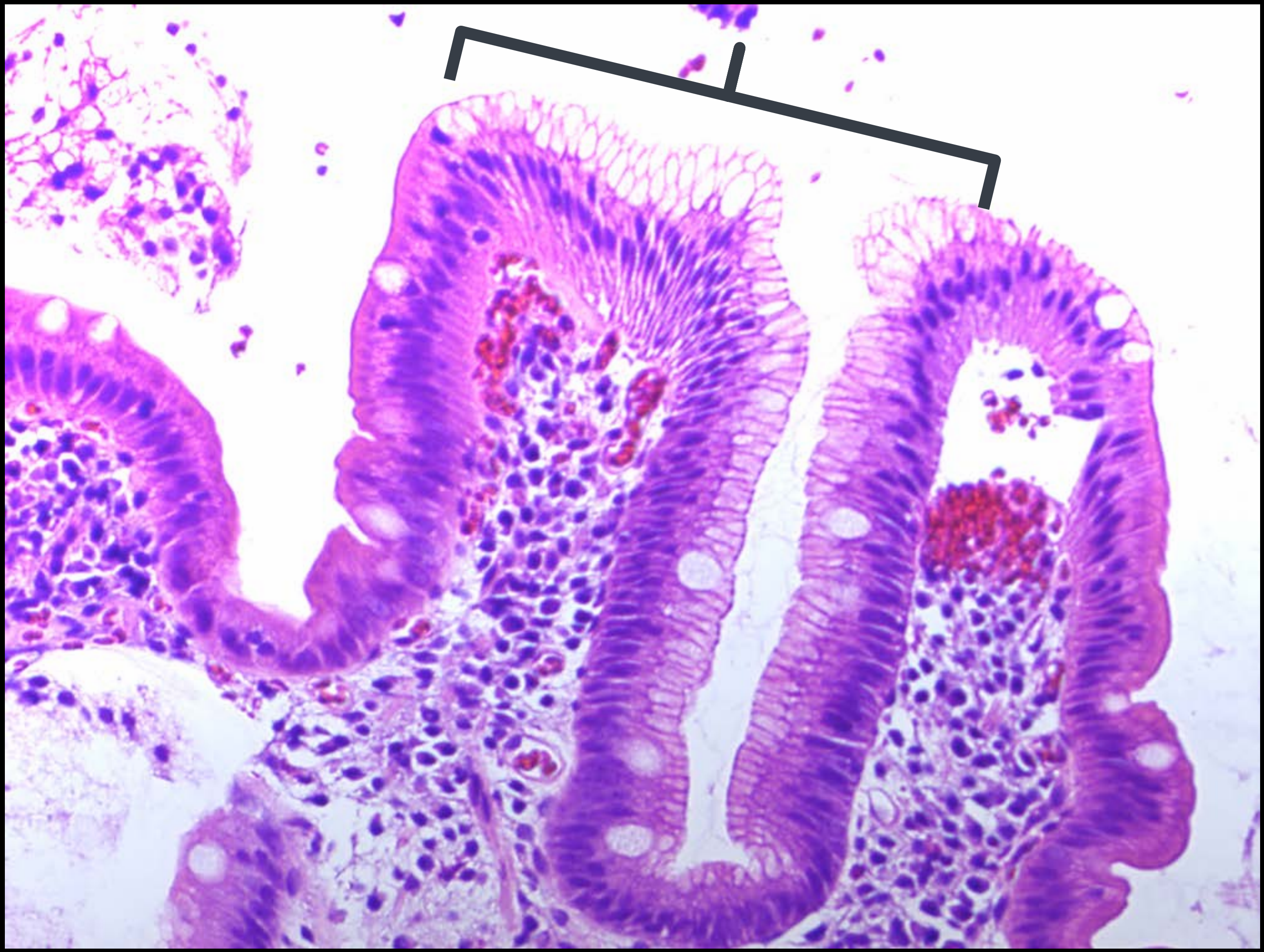

Foveolar mucin cells observed, usually on the tips of the villi

Grade 1: 1-2 villus tips involved

Grade 2: 3-5 villus tips involved

Grade 3: >5 villus tips involved

ENTEROCYTE INJURY
GRADE 0

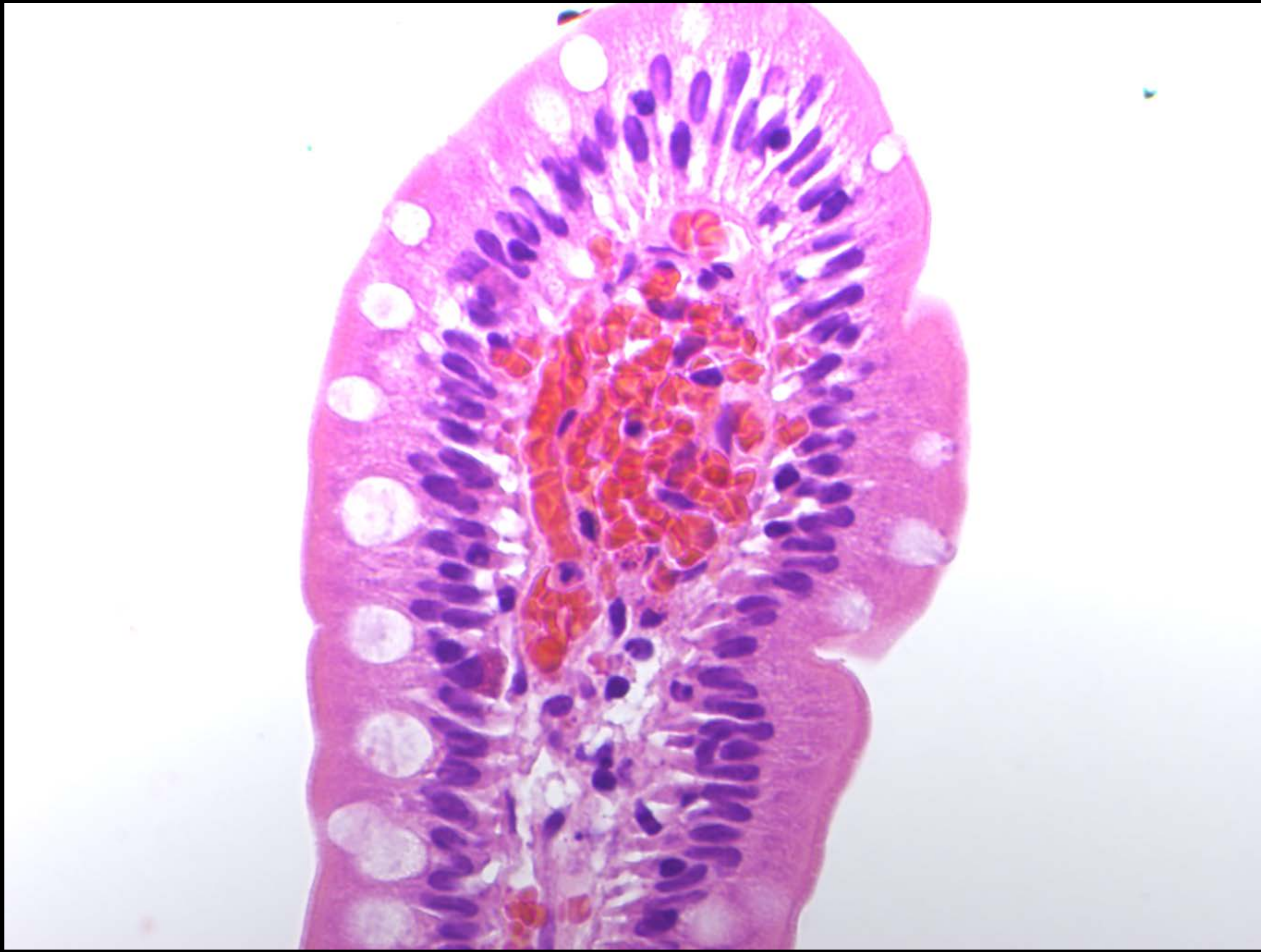

Grade 0: Majority of enterocytes (90%) show tall columnar morphology

ENTEROCYTE INJURY GRADES 1-2

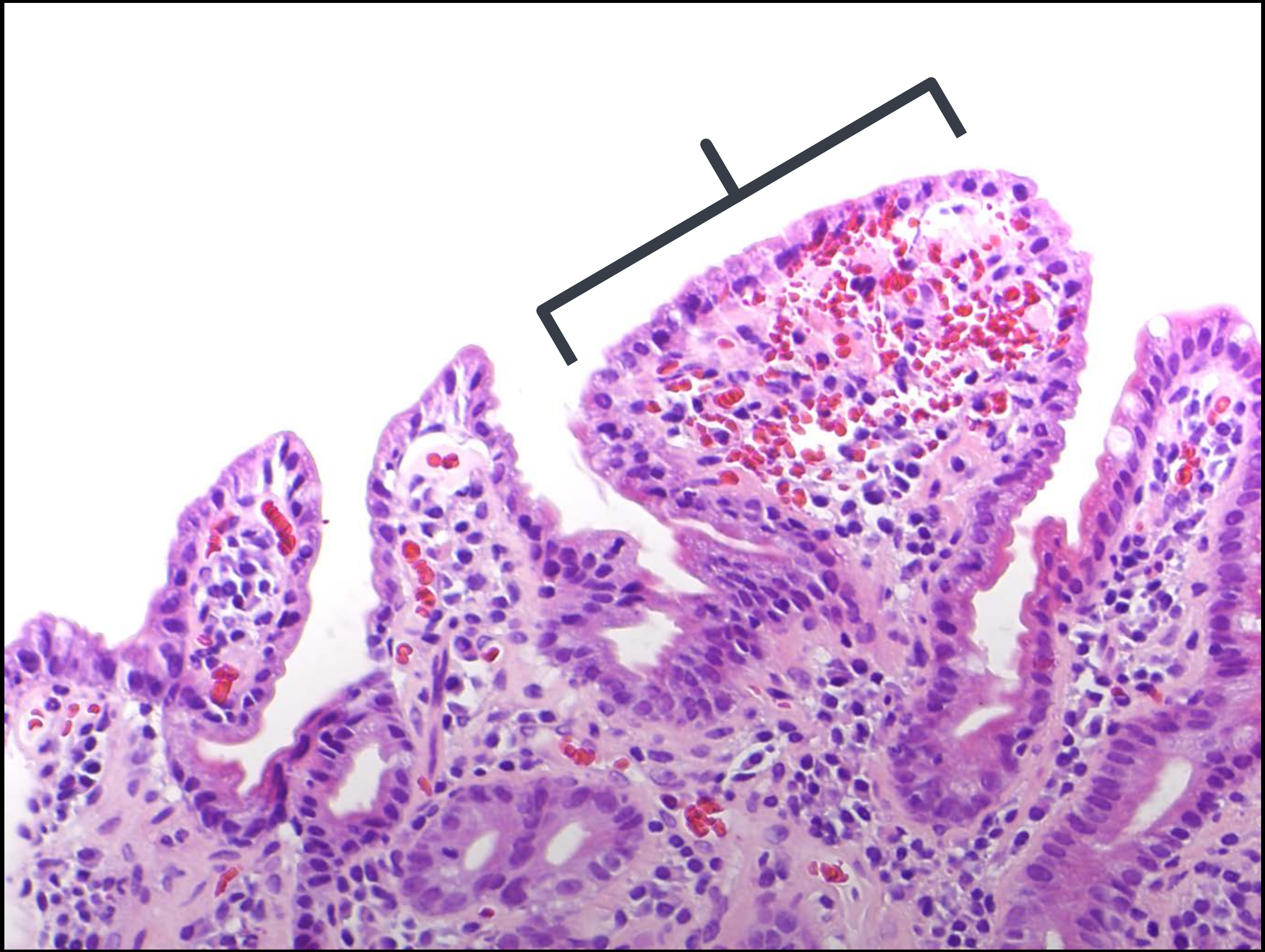

Enterocytes show short columnar (≤2:1 L:W ratio), cuboidal or flattened morphology

Grade 1: ≤ 50% of epithelial area

Grade 2: > 50% of epithelial area

ENTEROCYTE INJURY GRADE 3

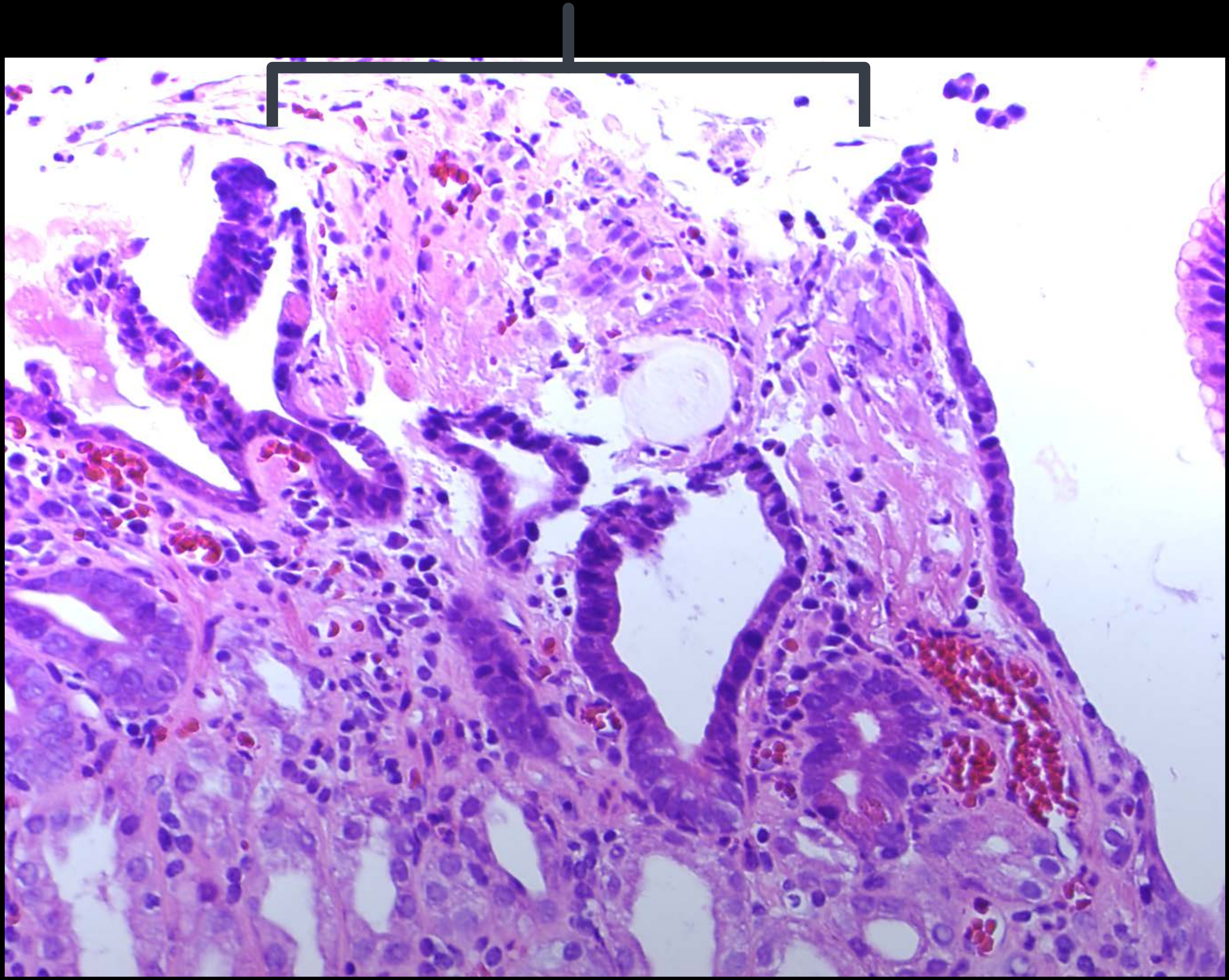

Any area of mucosal erosion or ulceration.

Note: presence of fibrino-inflammatory exudate and/or fibroblast proliferation help distinguish this injury from epithelial detachment artifact.

INTRAEPITHELIAL LYMPHOCYTES GRADE 0

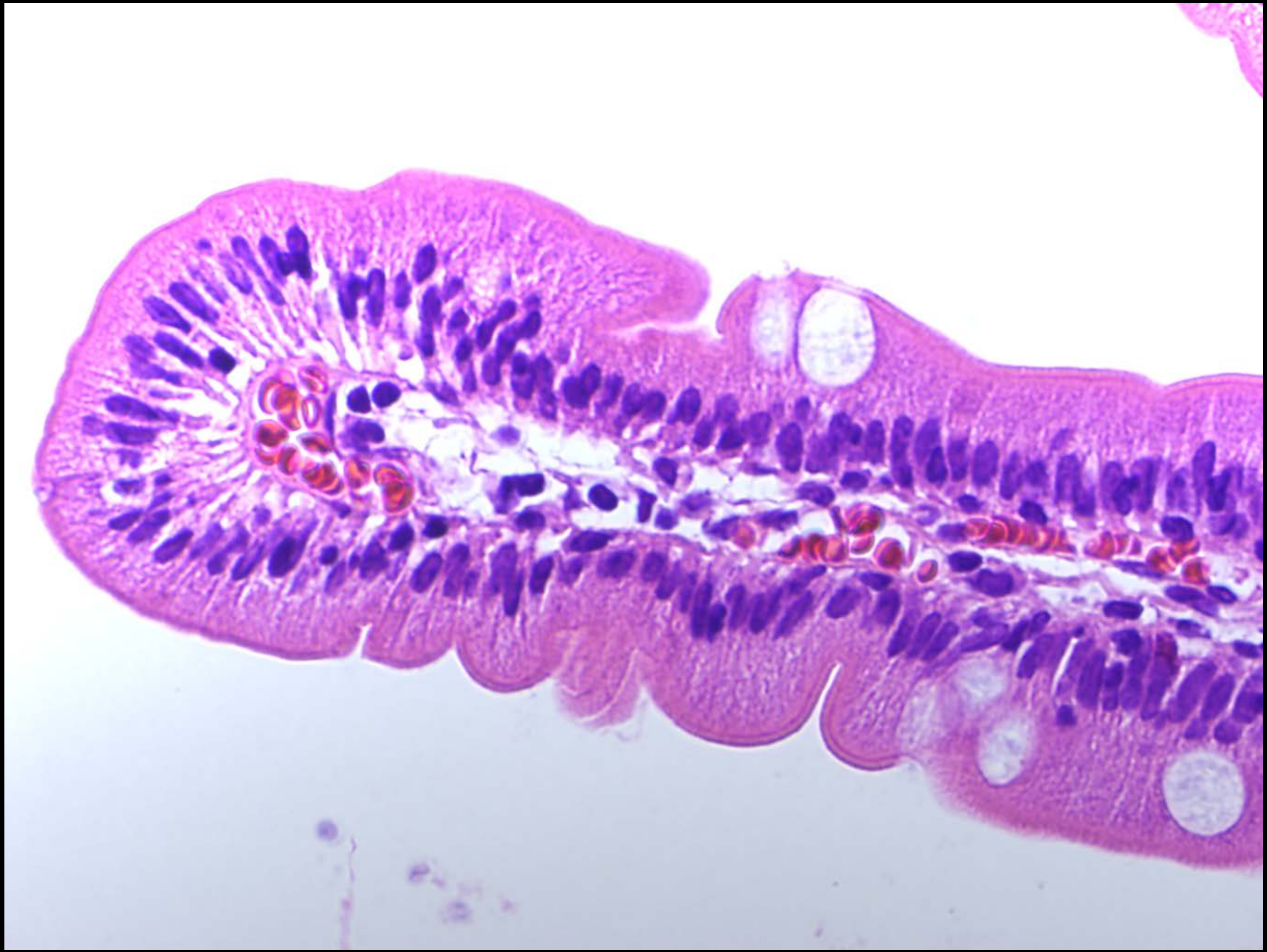

IEL ratio of lymphocytes to enterocytes does not exceed 1:5 (20%) in any area

INTRAEPITHELIAL LYMPHOCYTES GRADES 1 & 2

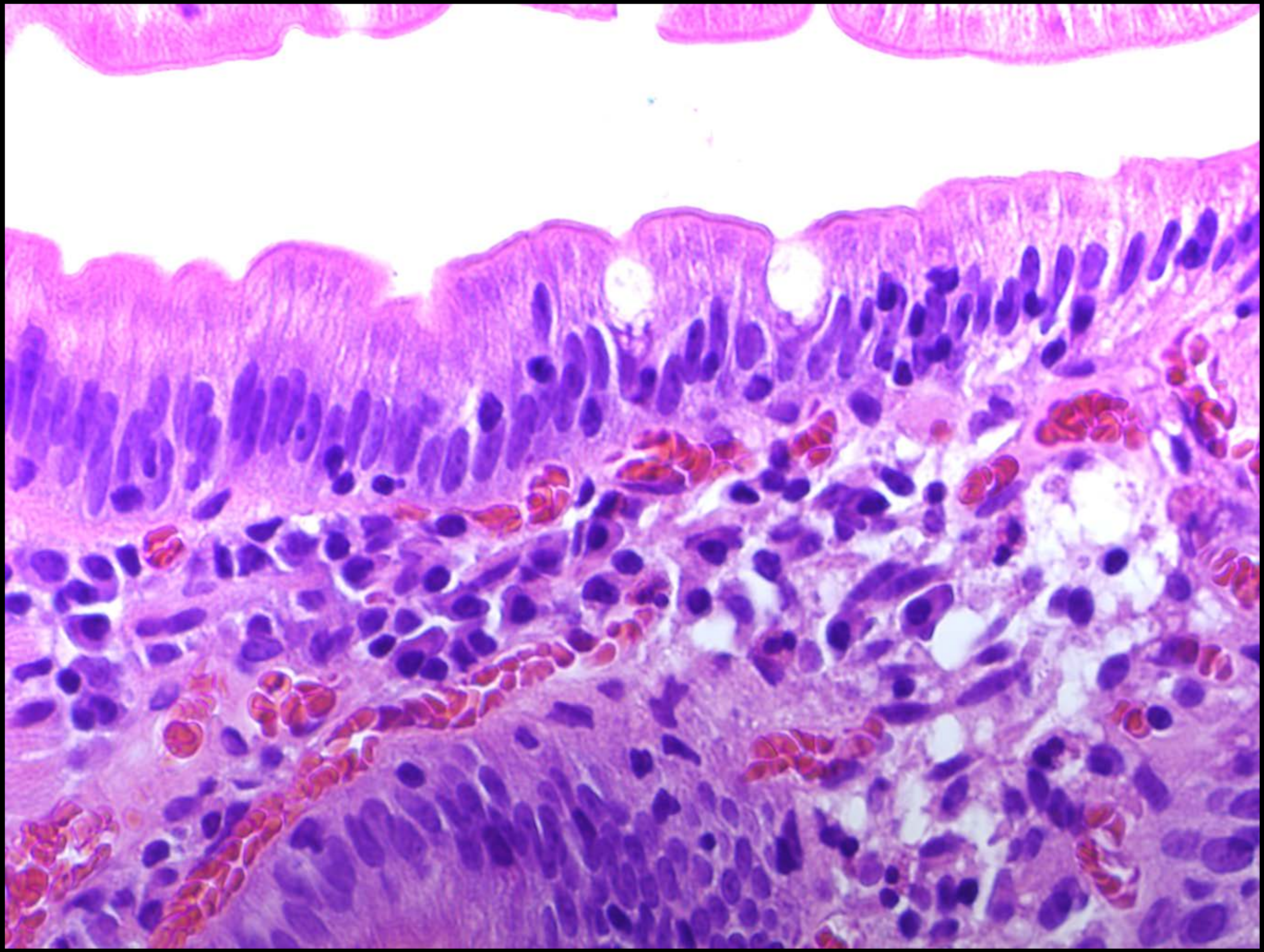

Grade1: Lymphocyte/epithelial ratio >20%, but < 50%, in less than 50% of mucosa

Grade 2: Lymphocyte/epithelial ratio >20%, but < 50%, in greater than 50% of mucosa

INTRAEPITHELIAL LYMPHOCYTES GRADES 3 & 4

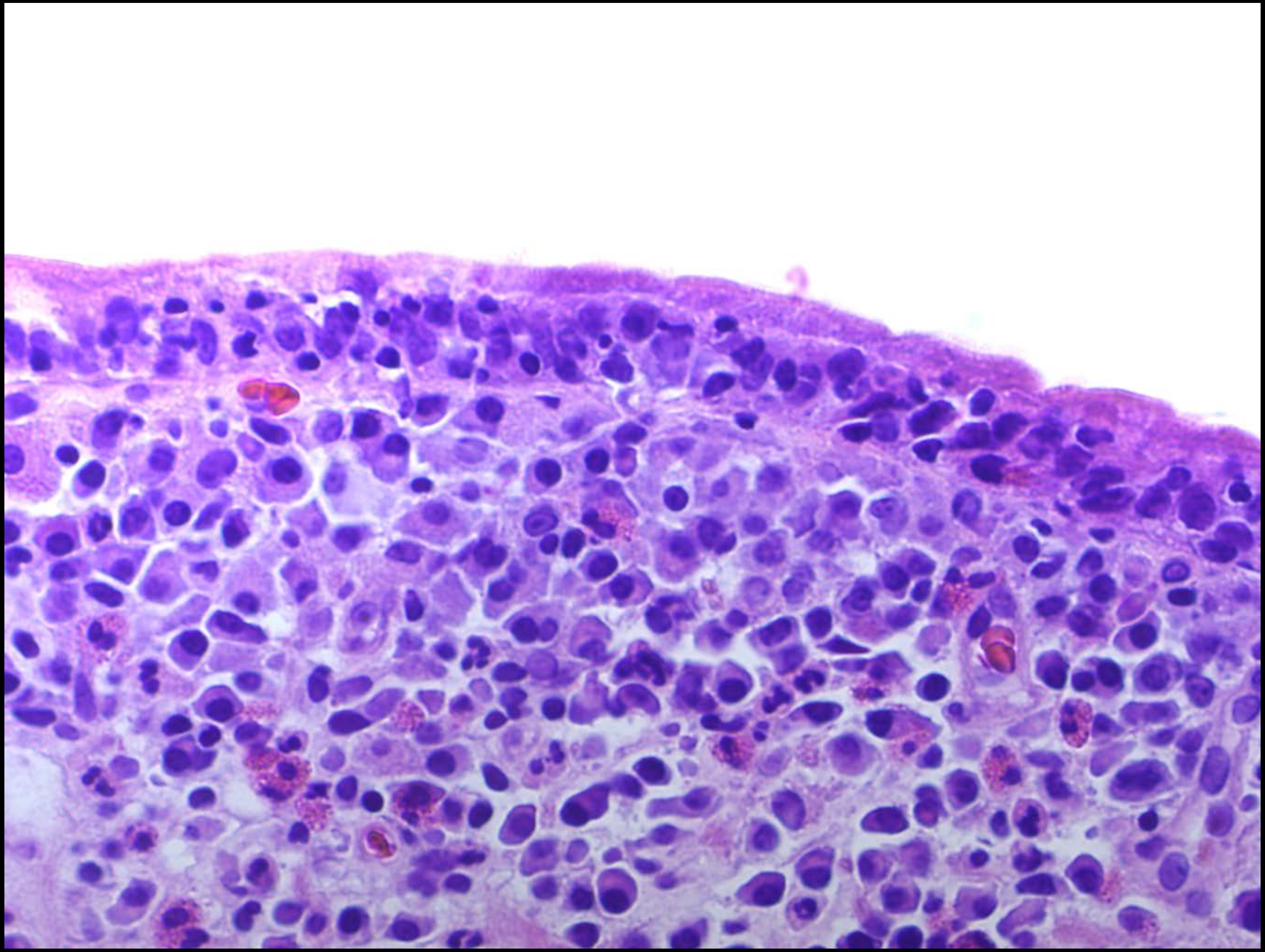

Grade 3:
Lymphocyte/epithelial ratio ≥50% in less than 50% of mucosa

Grade 4:
Lymphocyte/epithelial ratio ≥50% in greater than 50% of mucosa

CHRONIC INFLAMMATION OF LAMINA PROPRIA GRADE 0

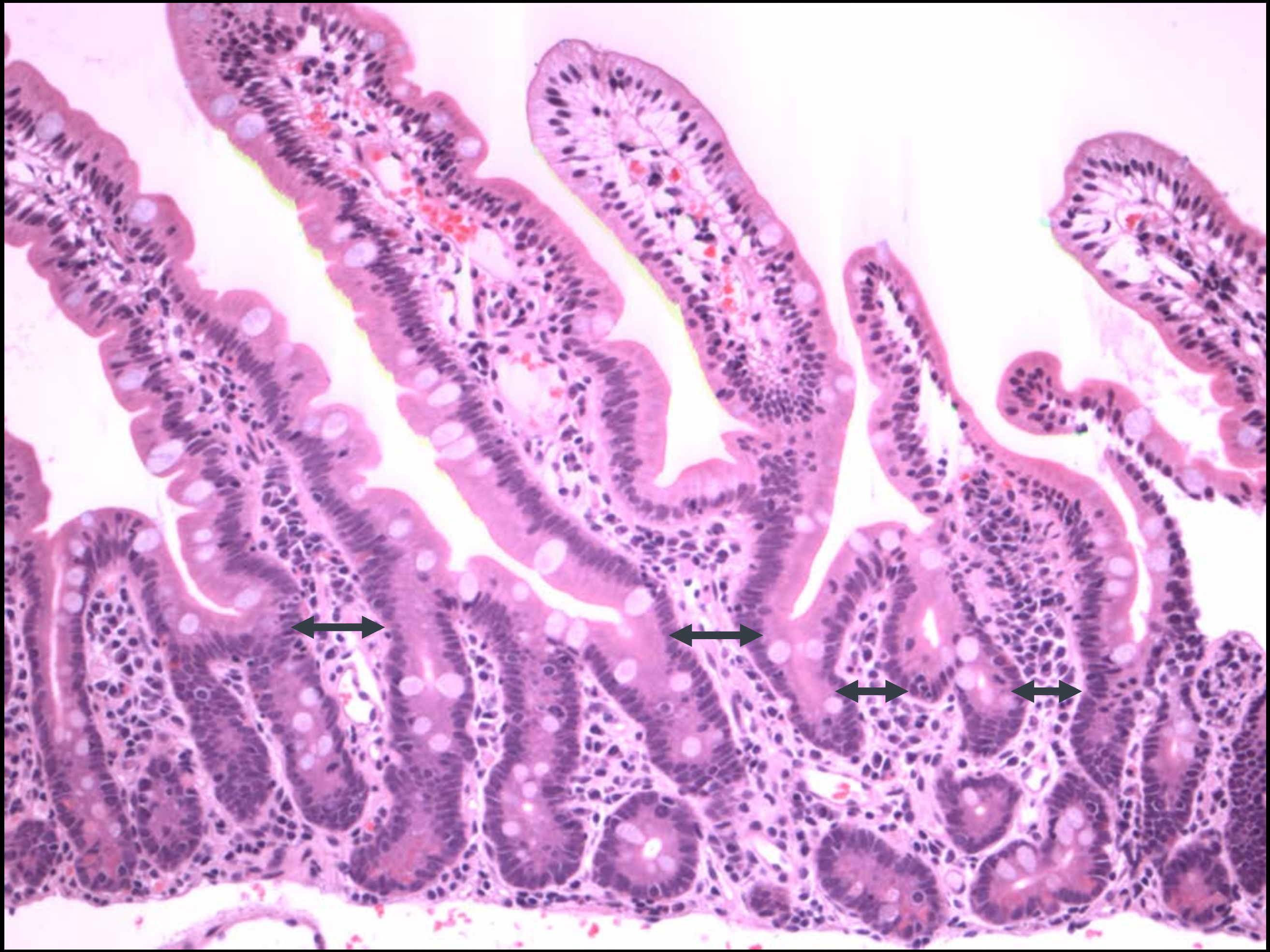

No qualitative increase in mononuclear inflammatory cells (MIC) in lamina propria. Majority of villus bases contain <3 MIC across, on average.

CHRONIC INFLAMMATION OF LAMINA PROPRIA GRADE 1

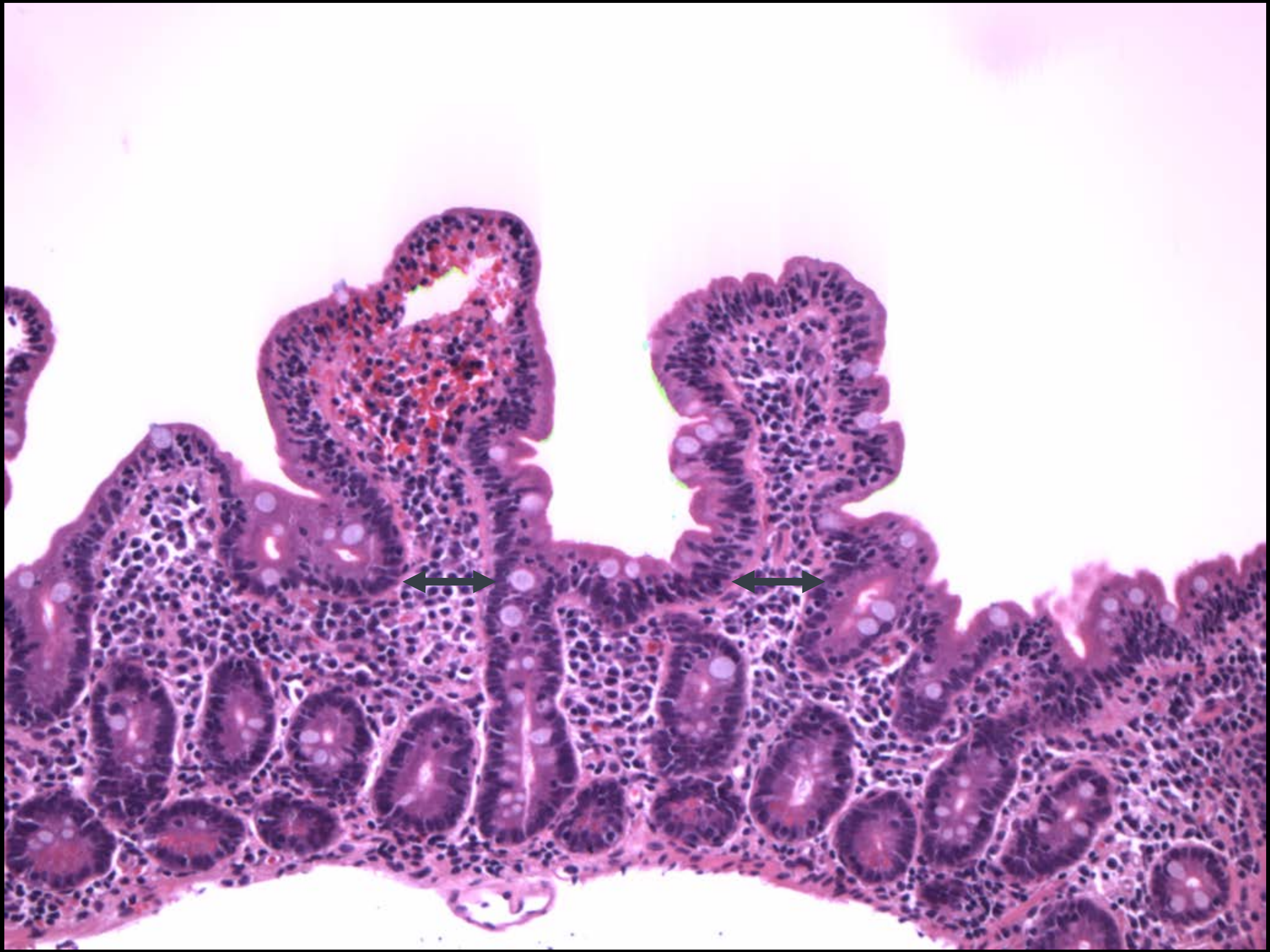

Increased MIC, based on villus base displaying 3-5 MIC across, on average.

CHRONIC INFLAMMATION OF LAMINA PROPRIA GRADE 2

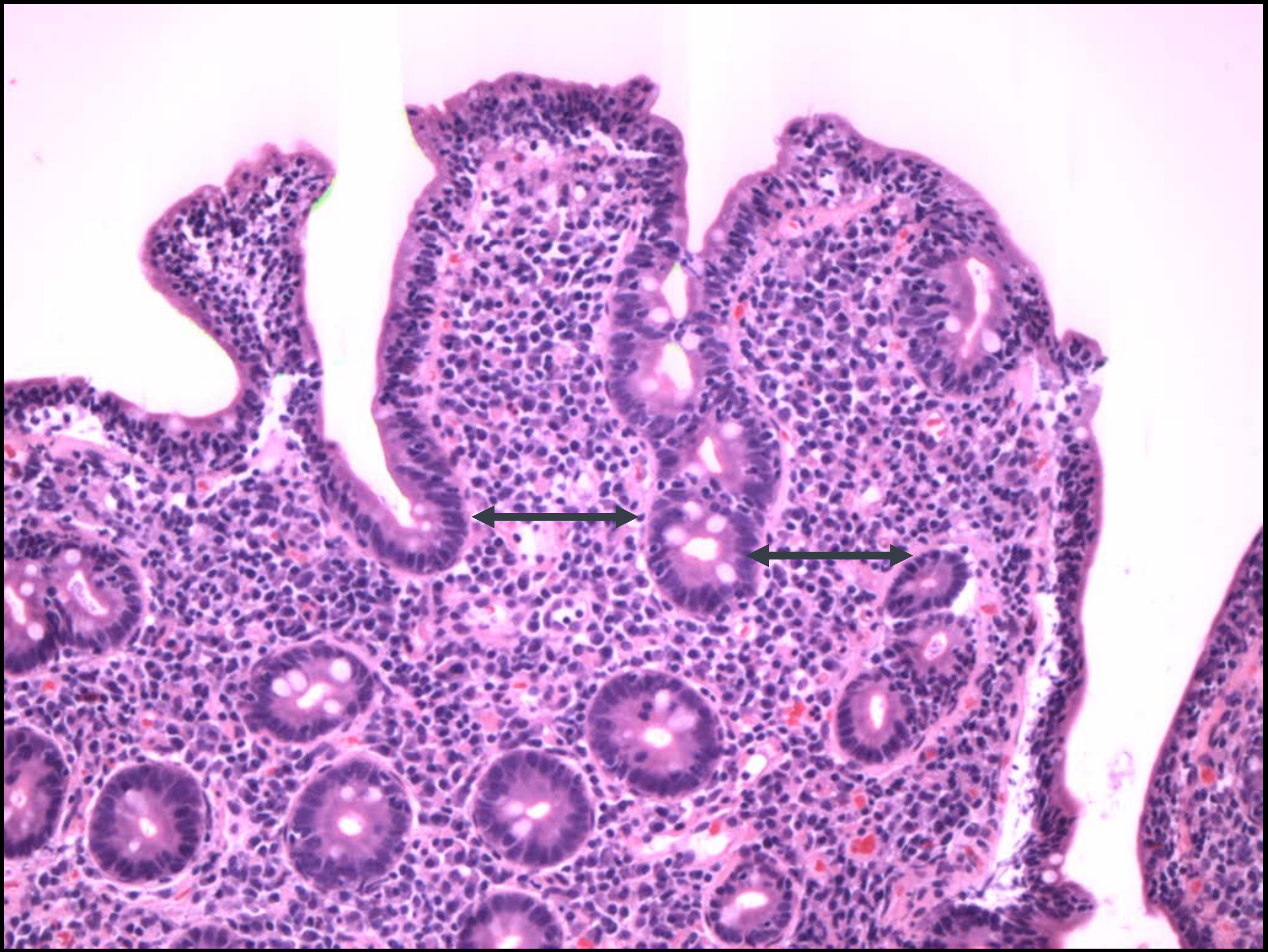

Increased MIC, based on villus base displaying 6-10 MIC across, on average.

CHRONIC INFLAMMATION OF LAMINA PROPRIA GRADE 3

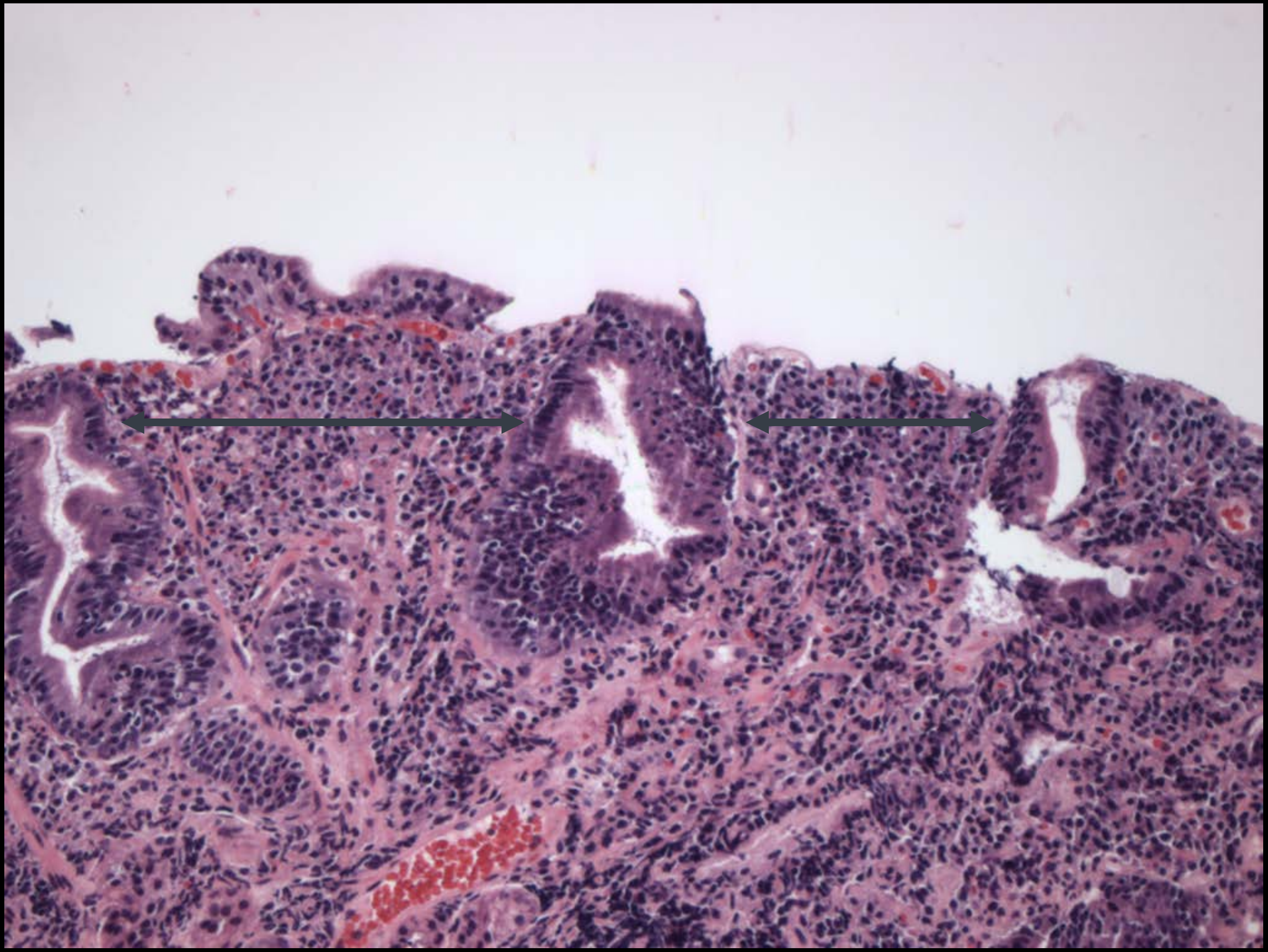

Increased MIC, based on villus base displaying >10 MIC across, on average.

Note: in cases of total villus atrophy, measure at surface of mucosa between crypt mouths

ACUTE INFLAMMATION
GRADE 0

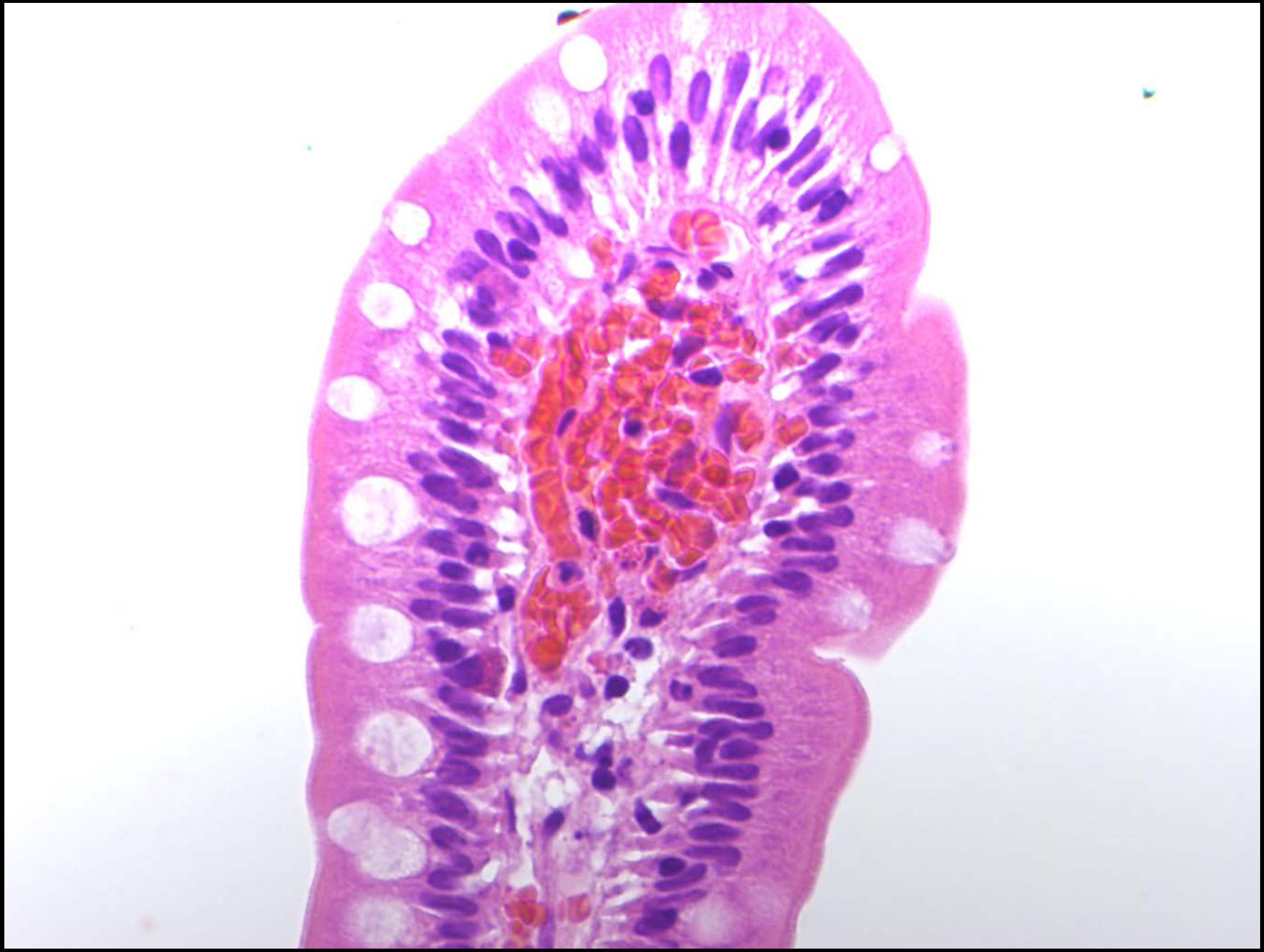

Grade 0: PMNs may be present in vessels or in lamina propria, but there is no intraepithelial infiltration (cryptitis, villitis)

ACUTE INFLAMMATION GRADES 1-3

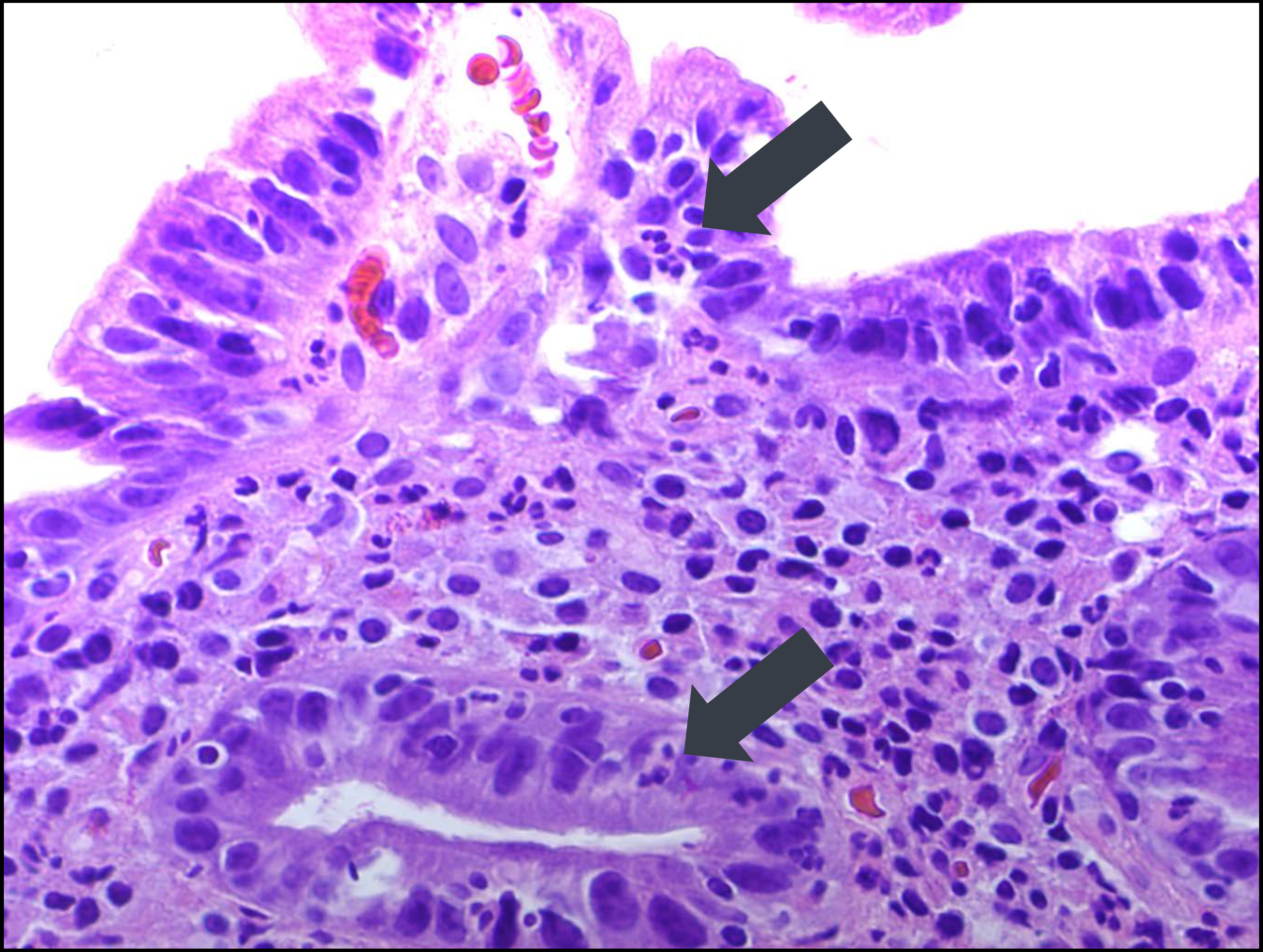

Grade 1:
1-2 foci of epithelial PMN infiltration or crypt microabscesses

Grade 2:
>2 foci of epithelial PMN infiltration or crypt microabscesses but ≤ 50% of mucosa involved

Grade 3:
>50% of mucosa involved by epithelial PMN infiltration

EOSINOPHIL INFILTRATION GRADE 0

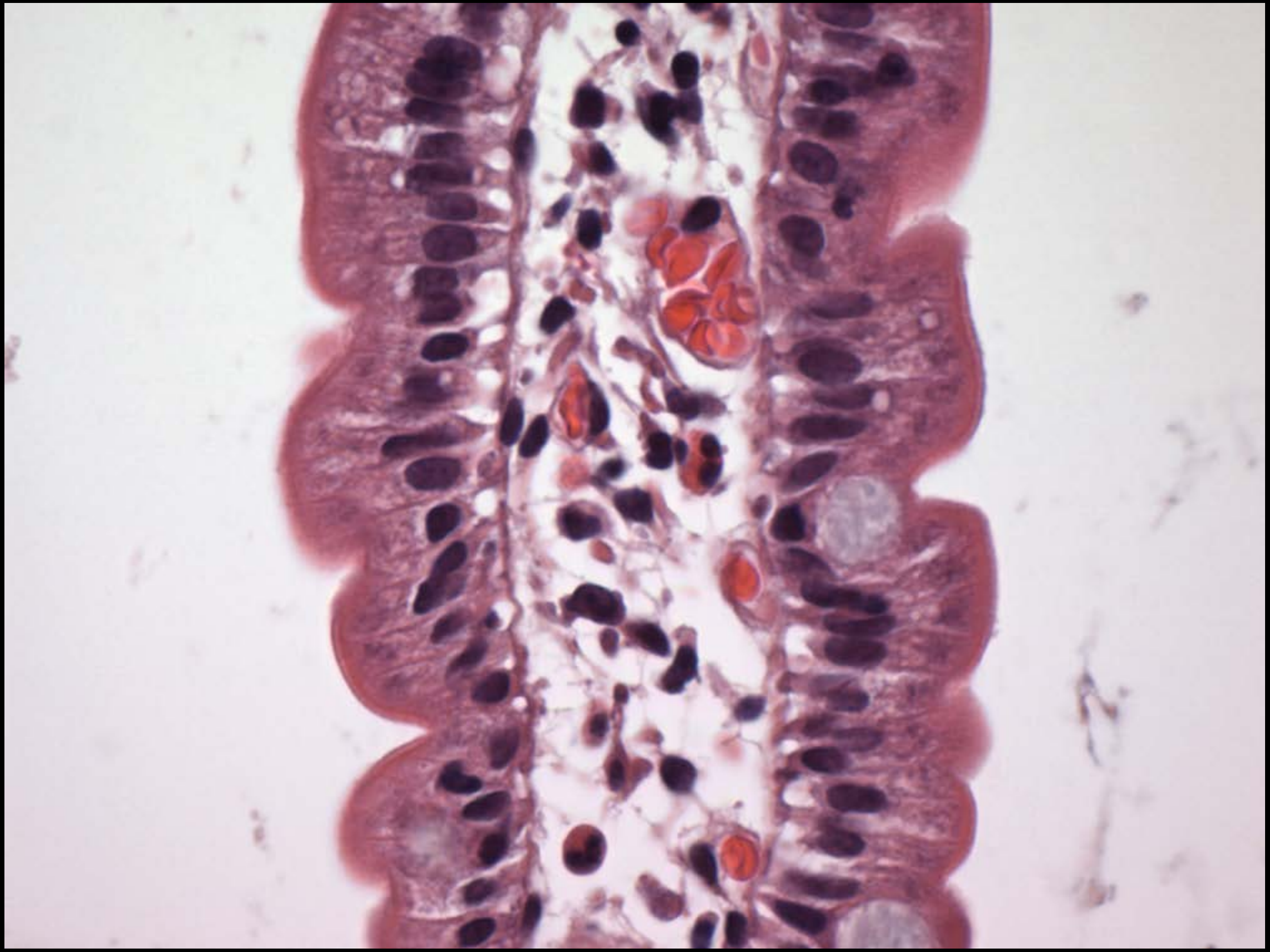

Grade 0:
No increase in eosinophils (highly scattered in lamina propria, no intravillus or intercryptal space with >5 eosinophils)

EOSINOPHIL INFILTRATION GRADES 1 & 2

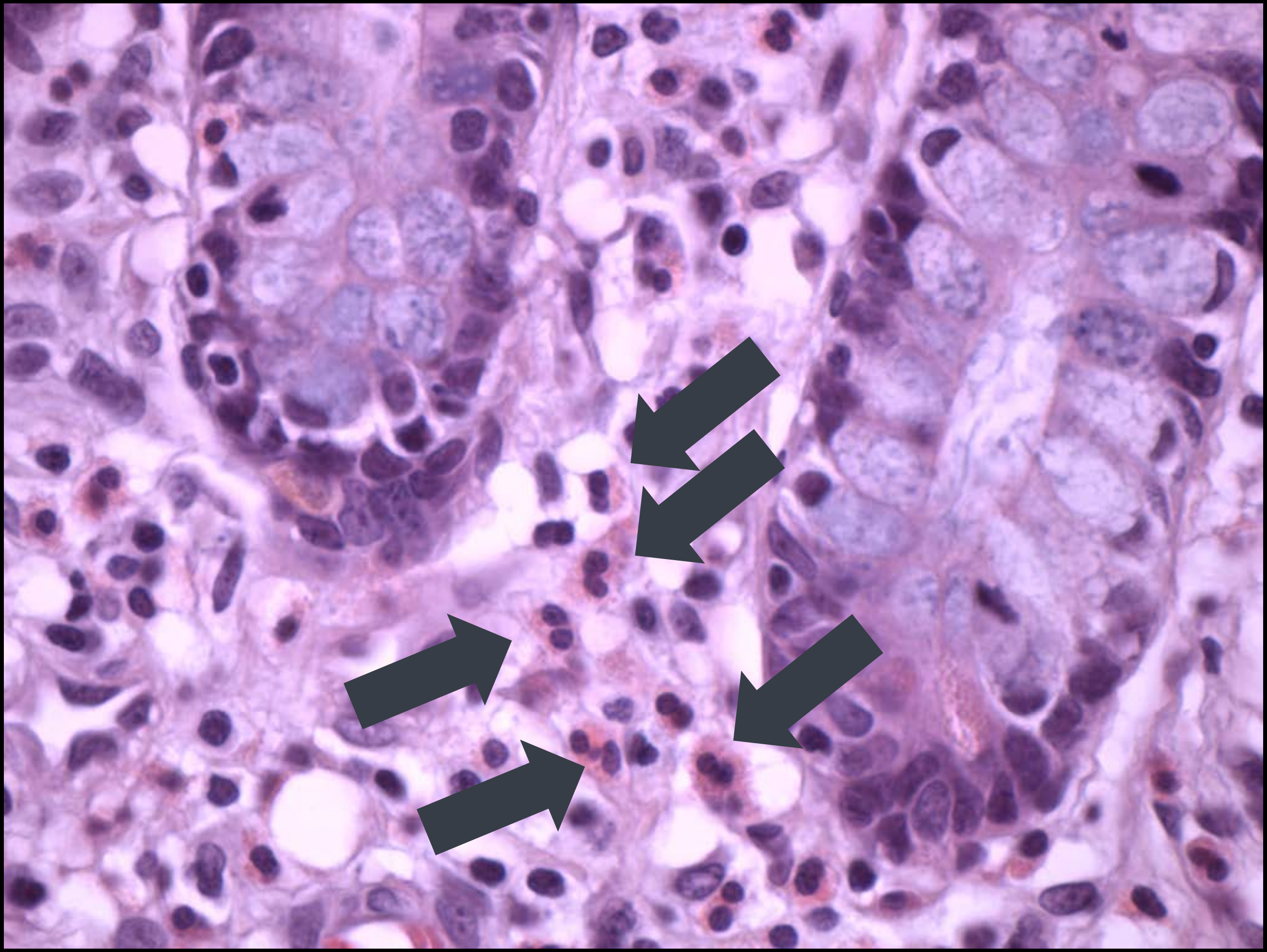

Grade 1:
Increased eosinophils (intravillus or intercryptal space with >5 eosinophils) involving ≤ 50% of mucosa, with no eosinophilic crypt microabcesses

Grade 2:
Increased eosinophils (intravillus or intercryptal space with >5 eosinophils) involving > 50% of mucosa, or up to 1 focus of eosinophilic epithelial infiltration or crypt microabcesses per mucosal fragment

EOSINOPHIL INFILTRATION GRADE 3

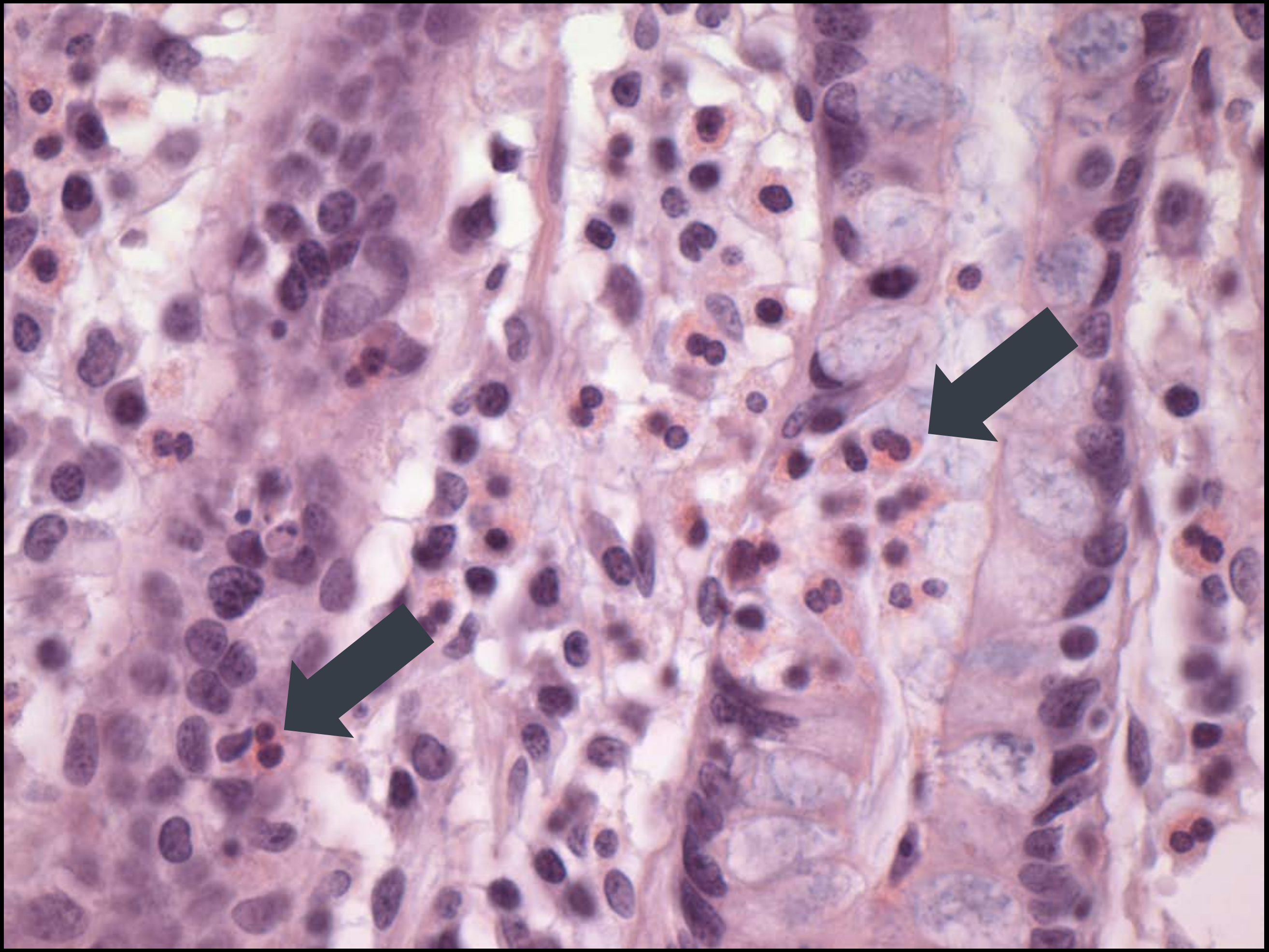

Grade 3:
>2 foci of eosinophilic epithelial infiltration or crypt microabcesses in any mucosal fragment

PANETH CELL DENSITY
GRADE 0

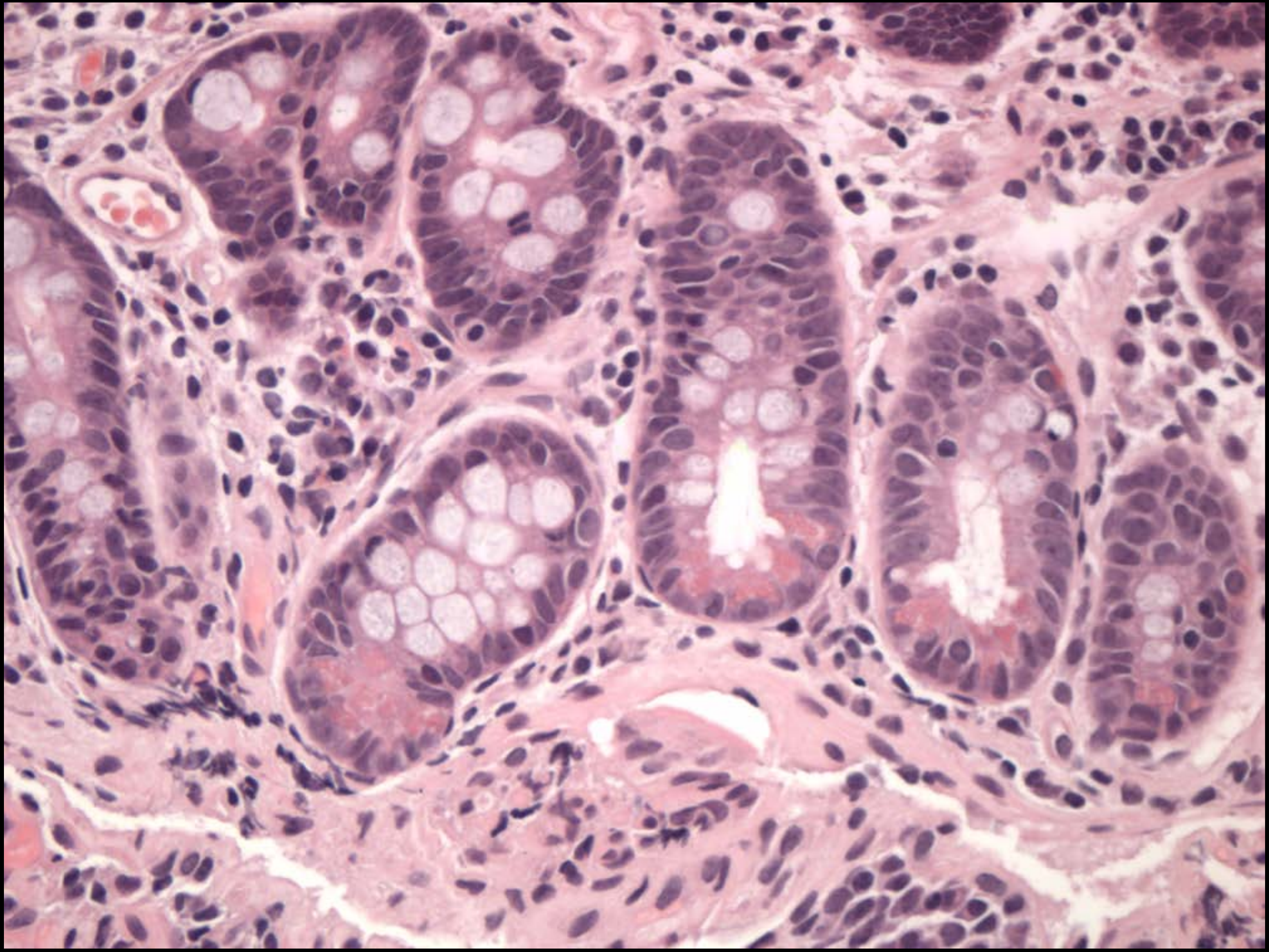

≥5 Paneth cells/ crypt base, on average

Note: only evaluate areas which crypt bases are well represented – avoid areas of complete transverse sectioning

PANETH CELL DENSITY
GRADE 1

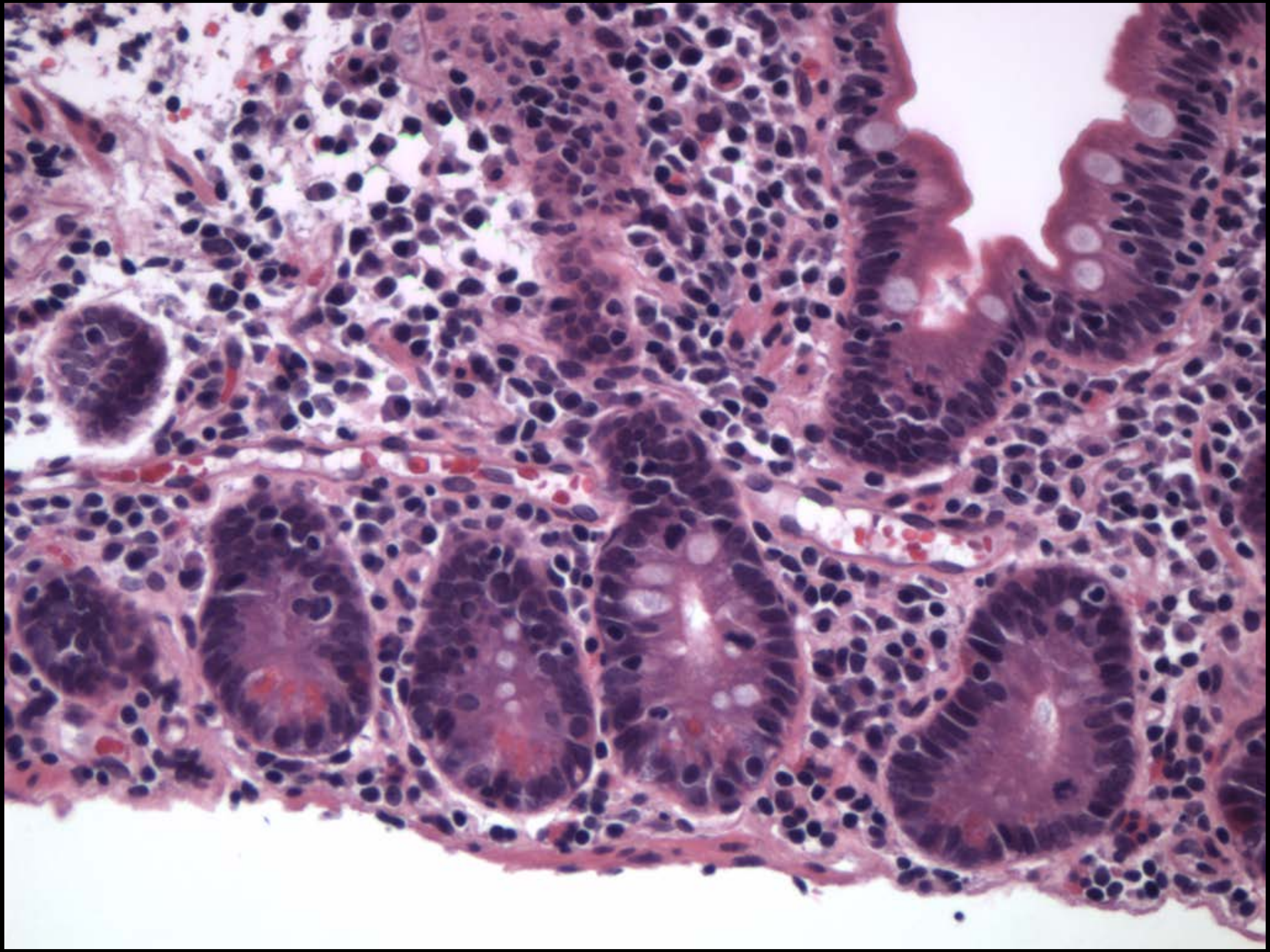

2-4 Paneth cells/ crypt base, on average

PANETH CELL DENSITY GRADES 2-3

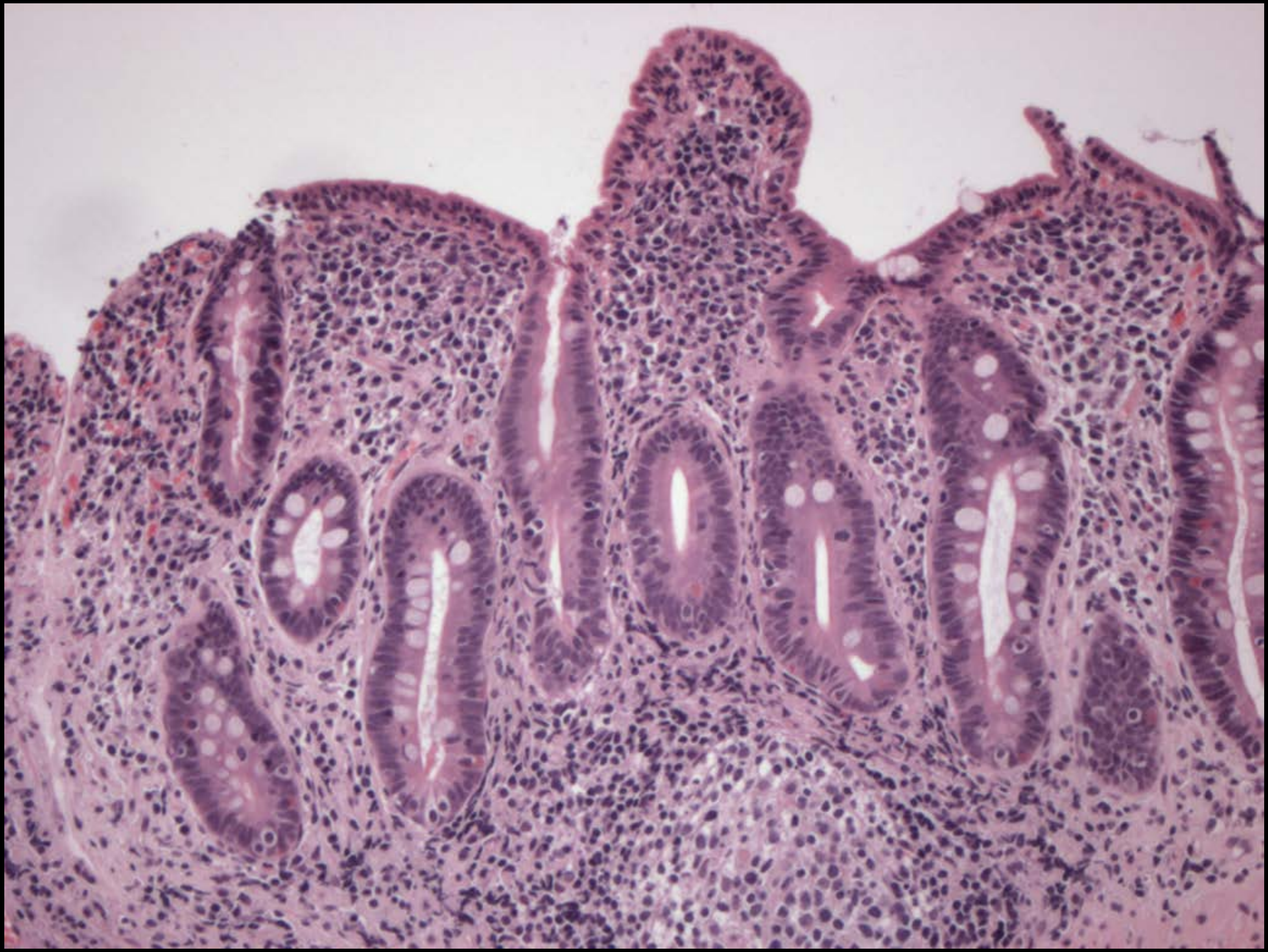

< 2 Paneth cells/ crypt base

Grade 2: seen in $\leq$50% of crypt bases

Grade 3: seen in >50% of crypt bases
