# Supplementary figures and images for: A novel histological index for evaluation of environmental enteric dysfunction identifies geographic-specific features of enteropathy among children with suboptimal growth
Source: PLoS Negl Trop Dis. 2020 Jan 13;14(1):e0007975. doi: 10.1371/journal.pntd.0007975 (PMC6980693; doi:10.1371/journal.pntd.0007975)

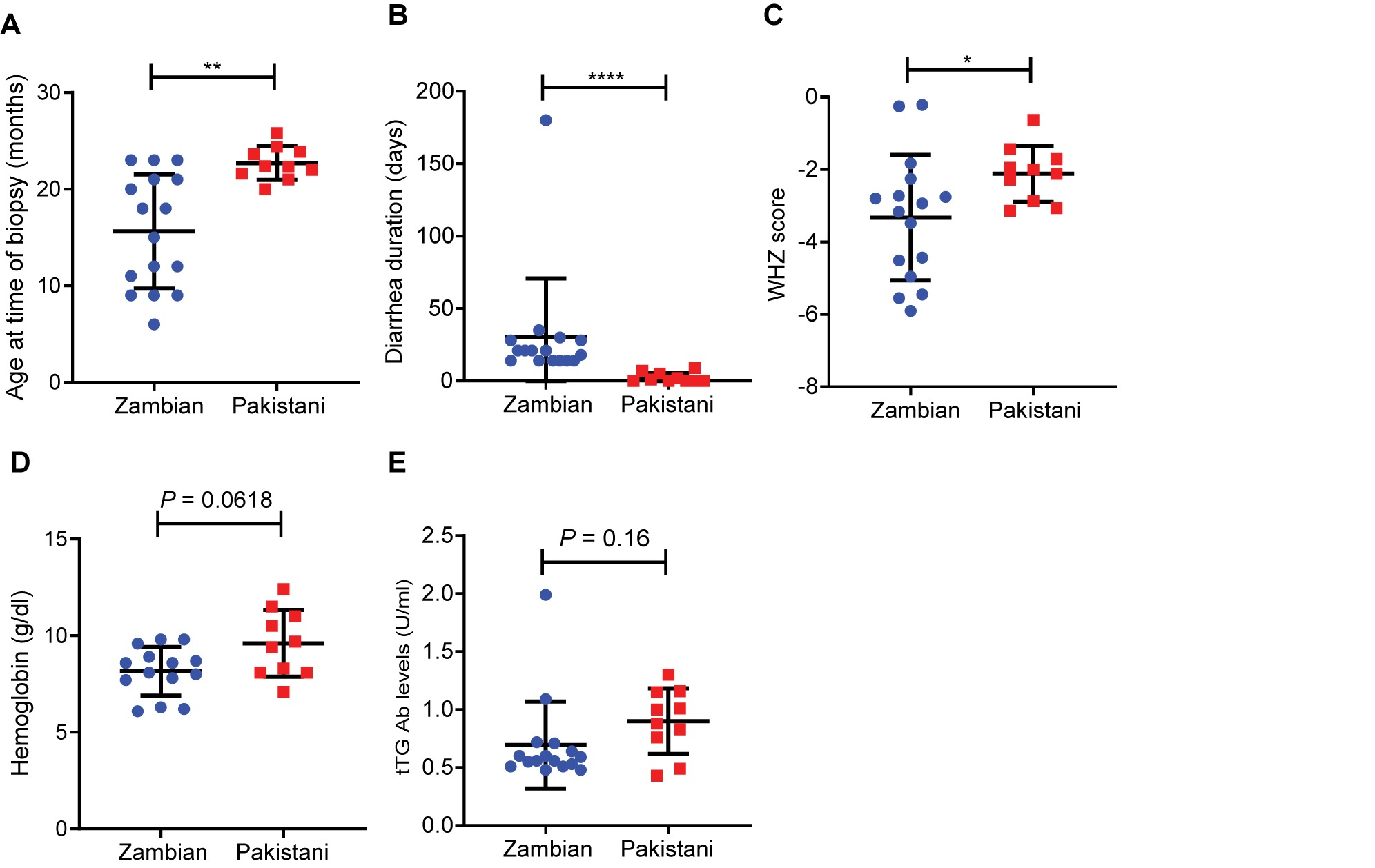

Supplement: S1 Fig — Due to differences in study design, the subjects in the Zambian cohort were (A) younger (p = 0.0012), (B) had diarrhea histories of longer durations (p < 0.0001), and (C) had borderline lower WHZ score (p = 0.047), Hemoglobin concentrations were marginally lower in the Zambia cohort (p = 0.062) and there was no difference in tTG antibody circulating concentrations (as a percent of upper limit of normal, p = 0.16). Statistical analysis was performed by Mann-Whitney tests. (TIF) [file pntd.0007975.s009.tif]

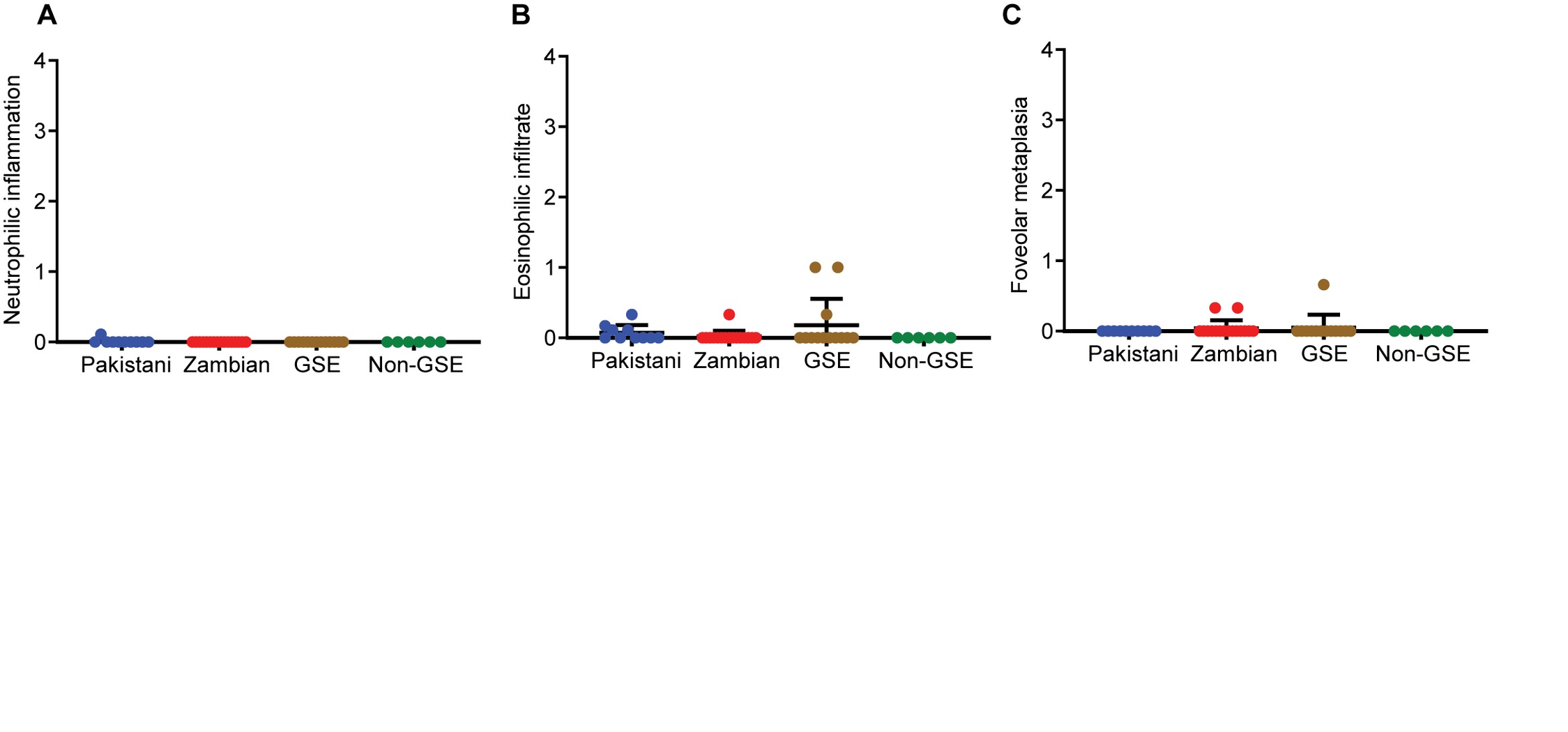

Supplement: S2 Fig — None of these cohorts had significant differences in (A) neutrophilic inflammation, (B) eosinophilic infiltration, and (C) foveolar metaplasia. Sample size: Pakistani n = 10, Zambian n = 16, GSE n = 13, control n = 6. (TIF) [file pntd.0007975.s010.tif]

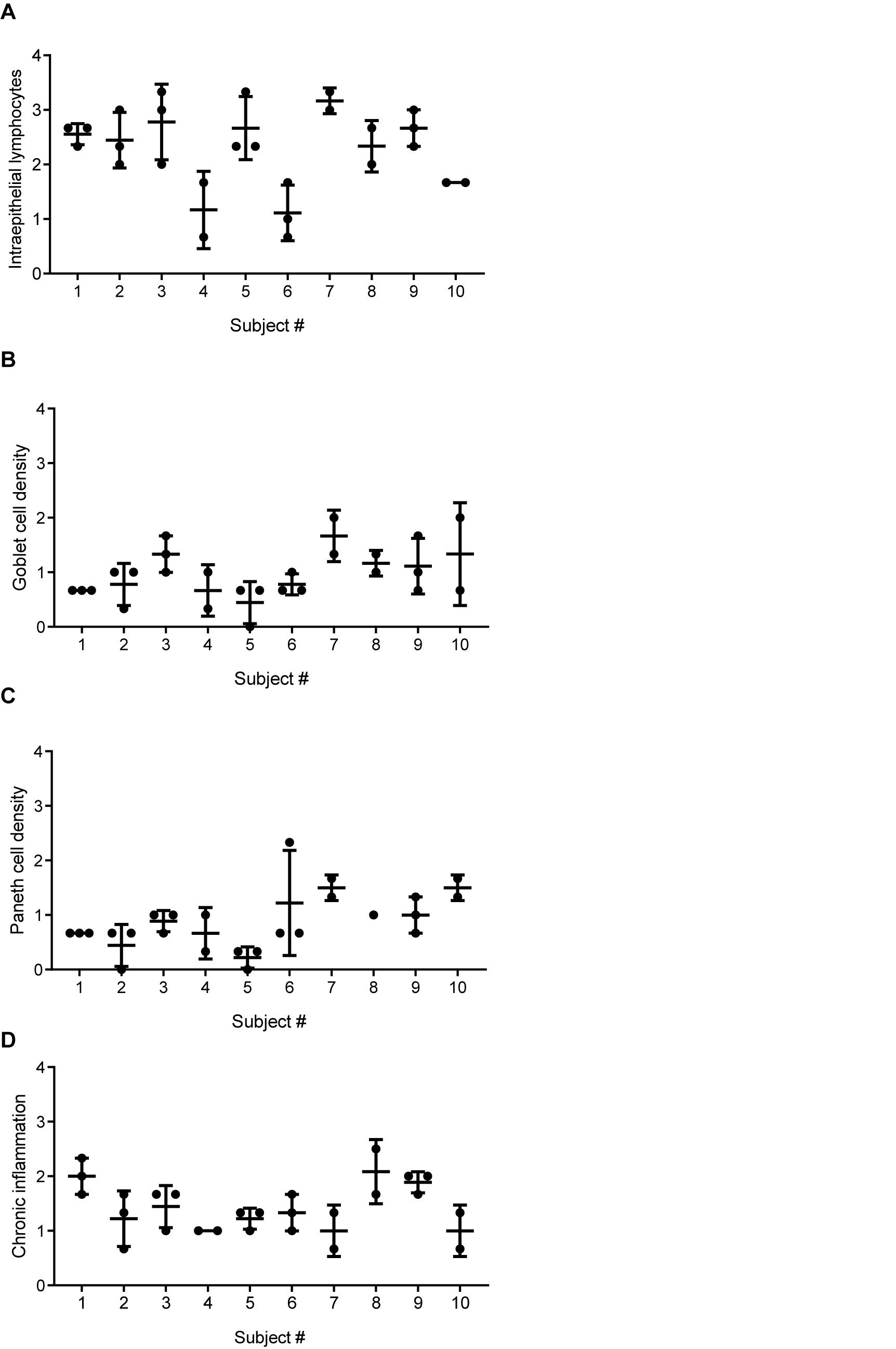

Supplement: S3 Fig — The scores for each individual biopsy for each subject were plotted for (A) intraepithelial lymphocytes density, (B) goblet cell density, (C) Paneth cell density, and (D) chronic inflammation. These parameters showed some within-child variation, but less than the scores for villous architecture and intramucosal Brunner glands. (TIF) [file pntd.0007975.s011.tif]
